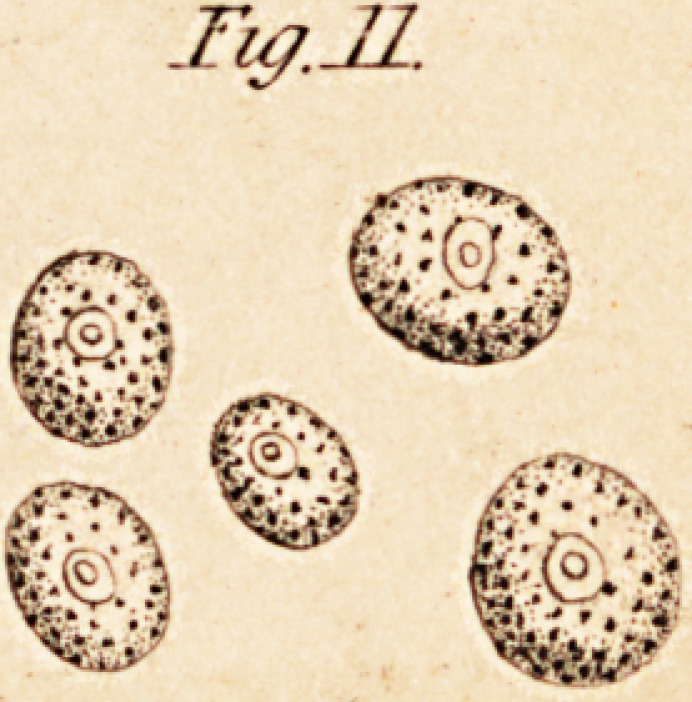# Discovery of a Remarkable Structure of the Brain and Nerves in Man and Animals

**Published:** 1838-10

**Authors:** 


### ART. VIII.

1. *Beobachtung einer auffallenden bisher unerkannten Structur des Seelenorgans bei Menschen und Thieren.* Von C. G. EHRENBERG, &c.—*Berlin*, 1836.

*Discovery of a remarkable Structure of the Brain and Nerves in Man and Animals.* By C. G. EHRENBERG. *With six Copper-plates, representing the Structure of the Brain in Man and thirty Genera of Animals.* Read before the Academy of Sciences of Berlin, October, 1833.—*Berlin*, 1836. Folio. pp. 57.

2. *Anatomie der mikroskopischen Gebilde des menschlichen Körpers.* Von Dr. JOSEPH BERRES, kaiserl. königl. ordentl. öffentl. Professor der Anatomie an der Wiener Universität.—*Wien*, 1837.—*Anatomia microscopica Corporis Humani.* Auctore Dre JOSEPHO BERRES, Professore publico ordinario in Universitate Vindobonensi, &c.—*Viennæ*, 1837.

*Microscopical Anatomy of the Tissues of the Human Body.* By Dr. JOSEPH BERRES, Professor of Anatomy in the University of Vienna.—*Vienna*, 1837. Folio. *Plates.* Part V. *On the Nervous Substance.*

3. *Beiträge zur Aufklärung der Erscheinungen und Gesetze des organischen Lebens.* Von GOTTFRIED-REINHOLD TREVIRANUS. Ersten Bandes, zweites Heft. *Neue Untersuchungen über die organischen Elemente der thierischen Körper und deren Zusammensetzung.—Bremen*, 1835.—Ersten Bandes, drittes Heft. *Nachträge zu den Beobachtungen des Verfassers über den innern Bau der Retina.—Bremen*, 1837.

*Contributions to illustrate the Phenomena and Laws of Organic Life.* By GOTTFRIED REINHOLD TREVIRANUS. Vol. I. Part II., *containing New Researches on the Organic Elements of Animal Bodies and their Combination.—Bremen*, 1835.—Part III. pp. 91-101. *A Supplement to the Observations of the Author on the intimate Structure of the Retina.—Bremen*, 1837. 8vo.; *with Plates.*

4. *Vorläufige Mittheilung microscopischen Beobachtungen über den innern Bau der Cerebrospinalnerven und über die Entwickelung ihrer Formelemente.* Von ROBERT REMAK. (*In Müller's Archiv. Jahrgang*, 1836. Heft II.)

*Provisional Communication of microscopical Observations on the intimate Structure of the Cerebro-spinal Nerves, and on the Development of their elementary Forms.* By ROBERT REMAK. (*In Müller's Archives, for the year* 1836. Part II.)

5. *Ueber den Verlauf und die letzten Enden der Nerven.* Von G. VALENTIN. (*In Nova Acta Cur. Nat., &c.* Vol. XVIII.)—*Breslau und Bonn*, 1836.

*On the Course and Terminations of the Nerves.* By G. VALENTIN. (*In Nova Acta Cur. Nat., &c.* Vol. XVIII.)—*Breslau and Bonn*, 1836.

6. *Ueber die Endigungsweise der Nerven in den Muskeln, nach eigenen Untersuchungen.* Von Dr. FRIEDRICH-CARL EMMERT, Privatdocenten an der Hochschule in Bern.—*Bern*, 1836.

*On the Mode of Termination of the Nerves in the Muscles.* By Dr. FREDERIC CHARLES EMMERT, Private Lecturer in the University of Berne. *With two Lithographic Plates.—Berne*, 1836. 4to. pp. 35.

7. *Beitrag zur mikroskopischen Anatomie der Nerven.* Von Dr. ERNST BURDACH, Prosector und Privatdocenten an der Universität zu Königsberg. *Mit zwei Kupfertafeln.—Königsberg*, 1837.

*Contribution to the Microscopical Anatomy of the Nerves.* By Dr. E. BURDACH, Prosector and Private Lecturer in the University of Koningsberg. *With two Copper-plates.—Koningsberg*, 1837. 4to. pp. 76.

8. *Elements of Physiology.* By J. MUELLER, M.D., Professor of Anatomy and Physiology in the University of Berlin, &c. *Translated from the German, with Notes, by* WM. BALY, M.D., Graduate of the University of Berlin, and Physician to the St. Pancras Infirmary.

*Illustrated with Steel-Plates and numerous Wood-Engravings.* Part III., *containing the Nervous System, and completing* Vol. I.—*London,* 1838. 8vo. pp. 848.

"Das Wissen wird im Suchen sich entfalten,"—Knowledge will unfold herself to him who investigates: such is the motto on the title-page of Ehrenberg's memoir. The career of the esteemed author is a commentary on it. By his patient and persevering researches he has unfolded a wonderfully complex organization in beings previously supposed to be little more than specks of homogeneous matter: nay, as Burdach remarks in the work the title of which we have placed above, he has even shown that certain dead masses of stone consist of antediluvian infusoria.

By the observations detailed in the memoir now under notice, Ehrenberg has certainly not unfolded everything that could be wished regarding the intimate structure of the brain and nerves; still he has cast such a blaze of light on the subject as has already served to guide to more extended and accurate knowledge.

Knowledge will unfold herself to him who investigates,—provided always the investigations be conducted in a proper way. We must enter upon the path of scientific enquiry, as in every other search after truth, in a true spirit of simplicity. Throwing aside all preconceived notions, we must be contented to see only what nature presents. In matters of pure science there is no occasion to hurry on, right or wrong, to some conclusion: it is much better, when we cannot obtain facts enough, either by the observation or interrogation of nature, to suspend our judgment. *No* conclusion is better than a wrong one; and, where judgment is suspended, it is more likely that greater efforts will be made to clear up the subject, than if ignorance were cloaked over by a conclusion of some kind. These reflections will be found to serve in some degree as the argument of the following historical sketch, the materials of which we have extracted from that contained in the first section of Ehrenberg's memoir.

As the structure of the brain and nerves is entirely a microscopical matter, so the history of our knowledge of the subject commences only with the time when the microscope was first applied to anatomical investigation: whatever was emitted on the subject previously was mere conjecture. Although it is curious to observe that what is now recognized to be a fact—viz. that the brain and nerves possess a tubular structure,—is an old and widely spread notion, still those who first promulgated the doctrine had no definite idea of the thing as it really exists. Some admitted it merely to tally with their own wild speculations regarding the function of the brain and nerves; others, though they acknowledged they could not assert the thing as an observed fact, still subscribed to the opinion.

Though the history of our knowledge of the intimate structure of the brain and nerves commences with the first application of the microscope to anatomical purposes, we encounter at first rather errors committed by the employment of that instrument than any important observations made. It is with the microscope as with the eye itself: it is not enough that a man see with it,—he must observe with it. From inattention to this, perhaps, rather than from the badness of the instruments employed, imperfect as they confessedly were at first, arose the errors on the subject, not only of the structure of the nervous substance, but of all other parts of the body. "Hence," says Burdach, (p. 4,) "it may be affirmed that Monro would have come to the same conclusion that he did, even though he had had a microscope by Frauenhofer; and that, on the other hand, Ehrenberg, even with Della Torre's imperfect glass lenses, would have made out the structure of the nervous substance as he has now done." Even at the present day we are not unfrequently met by ill-observed facts, or assertions that the enquirer has not been able to see what others have unequivocally observed.

Every tissue must be examined in as unchanged a state as possible. Alteration by putrefaction, or any other reagent, is sometimes a means of investigation,—a means of leading us to discover the true and natural state of things; but the state of any substance, as changed by such reaction, ought never to be taken into account when the structure as it exists in the living body, comes to be the question. How far, then, must those physiologists have erred who imagined, as Ehrenberg remarks, (p. 37,) that they could discover the true structure of organic substances, too delicate to be examined by them in the natural state, after having been boiled, hardened in spirits, or dried! It is against the abuse, not the use, of reagents in our minute anatomical enquiries that we speak: we therefore consider that Burdach has done well in studying the appearances which the nervous substance presents under different influences, chemical and mechanical, at different ages, after different kinds of death, and at different periods after death, as also in the morbid state. Valentin, too, has investigated the subject more or less in the same way; and Remak has paid particular attention to the appearances presented by the nervous substance at different ages.

An opinion prevails that high magnifying powers are indispensable for minute anatomical investigations: but this we know, that, by properly displaying the substance to be examined, and with a practical knowledge of and dexterity in manipulating on the part of the examiner, not only the structure of the nervous substance, but also many other structures, may be very well demonstrated by inferior magnifying instruments; by much lower powers than a person unacquainted with the subject might be led to imagine. A simple lens, magnifying 150 to 200 diameters, will show very distinctly parts, to see which properly it is often pretended a compound microscope of high power is indispensably necessary. Globules much more minute than those of the blood can be distinctly seen with such powers; and it is to be remembered that it does not require so high a magnifying power to see a fibre or tube as to see a globule of the same diameter. We wish to insist upon this point, and to show that it is not the extent of magnifying power alone which assists the microscopical enquirer. Without properly isolating and displaying the object to be examined, to increase the magnifying power only leads to error. Farther, we venture to assert that the exclusive employment of the compound microscope is not good. On account of its affording a higher magnifying power with a larger field, and enabling us to make exact admeasurements with great facility, it is of the utmost value for the examination and admeasurement of what has been already displayed by dissection under the simple microscope. The compressing instruments, so much used of late, can never serve as a perfect substitute for such dissection. Compression is a very valuable means in many cases, but in others it distorts the thing examined, or destroys it, and prevents it from being seen altogether.

As by the compound microscope we see merely the inverted image of the object, it is a thing next to impossible to manipulate under it. Emmert (p. 8,) appears to have done so: his manipulations, however, must have been very imperfect. In regard to displaying the primitive parts of the substance of the brain and spinal marrow, of ganglions, and the like tender structures, Burdach says (p. 14) that tearing asunder the parts by means of knives or needles is not applicable; "as such an operation, even when performed by the most steady hand, produces too great confusion and destruction of parts: it is, on the contrary, perfectly adapted for the demonstration of the primitive fibres of the peripheral nerves, as the latter preserve in the recent state a considerable degree of firmness." All this is true, if the simple microscope is not used first for the purpose of displaying the parts. Under the simple microscope, even with a magnifying power of 150 or 200 diameters, (though 100 diameters will in general suffice,) it is possible to display the most tender structures: if tearing asunder with fine needles be too rude and violent a mode, the parts may yet be separated and displayed by gentle touches, and by patiently agitating the water. The mere circumstance of putting the object under examination into motion frequently gives us valuable information. In displaying the part under the simple microscope, we can observe what changes take place during the process, and at the same time we can guard against the confusion which is so apt to occur; seeing that, in the most minute state of the manipulation, the eye guides the hand. We hold the use of the simple microscope in this way an indispensable accompaniment of the compound microscope, and we can easily trace many of the oversights of physiologists to the neglect of it.*

But to recur to our historical sketch:

Malpighi's microscopical examinations were confined to boiled brain, which he compared to a pomegranate full of seeds. In these he found glands and vascular saccules, because, says Ehrenberg, (p. 32,) he looked for them; having the idea that all the viscera possessed a glandular structure. After Malpighi, Leeuwenhoek, who may be considered the founder of microscopical anatomy, investigated the structure of the nervous substance, about the end of the seventeenth century; and, though some of the results he came to be not entirely discordant with reality, (less so even

* As an example of this may be adduced the fact of Purkinje and Valentin having searched in vain for the germinal vesicle in the mammiferous ovum, and at last detected it only on again resuming their investigations after learning that Coste, of Paris, had affirmed he had seen it. But, even after they found it, these acute investigators missed observing the spot on its side; a part which, in Germany, was shortly after pointed out by Wagner. In contrast with all this, we can state the fact that, in this country, about the same time the observations were made on the continent, the germinal vesicle of the mammiferous ovum was discovered, the spot on its side noticed, and a complete description with delineations of the thing given, by Mr. Wharton Jones, whose observations, we have the means of knowing, were made by the assistance of an ordinary simple microscope only, the highest power of which magnified about 200 diameters. No compression was used, but the parts simply displayed and dissected under the miscroscope, with common sewing needles stuck into wooden handles.

than the opinions which until lately prevailed on the subject,) still they are too imperfect to serve as a basis for any physiological deduction. A second series of investigations of the structure of the brain, which he instituted thirty years later, when, as he himself says, he was an old man, is absolutely of no value; as, instead of recent brain, which he had first employed, he took for the object of his second examinations dried brain. Leeuwenhoek was more successful in his investigation of the structure of some of the nerves. As early as 1674, he examined the structure of the optic nerve in the ox, and found it to consist of a filamentous substance, like vessels, filled with slowly moving globules. In the optic nerve of the horse he found globules similar to those larger and transparent ones which he had observed in the brain in the vicinity of the spinal marrow. In 1715, he announced that he had just discovered that the muscular nerves of animals are composed of from forty to twenty filaments; that not only are the individual nervous filaments hollow, but all are provided with many cavities. This assertion, which he put forth as being possibly subject to error, he confirmed, in 1717, by a direct observation of the cavities of the nerves in a cow. There is not in the nerves, according to him, any perceptible motion of fluid or a circulation. Here and there he found the spinal marrow present a structure similar to that of the nerves, but always very confused and obscure.

After Leeuwenhoek, Della Torre, Monro, and Fontana prosecuted the enquiry; but, unfortunately, though they examined the self-same substance by similar means, they arrived at contradictory results; a circumstance which materially retarded the farther development of the subject by bringing discredit on the microscope. Of the three, Fontana's observations come nearest the truth.

The lenses with which Della Torre made his observations were small unpolished glass-globules, prepared by himself. Having compressed the substance of the brain to be examined between plates of mica and glass, every organic texture was destroyed. He therefore represented the brain as a pulpy mass, consisting of globules suspended in a clear viscid fluid, and arranged here and there in rows. This view of the subject was supported by Prochaska, who differed only in considering the medium in which the globules were contained a delicate cellular tissue, and the nervous globules of equal size throughout; whereas Della Torre described them as being of different sizes in different parts of the brain and nerves.

Monro, ignorant of the mode of using the microscope, made his observations whilst the direct rays of the sun were transmitted through the substance under examination. The consequence was, as every one at all acquainted with the microscope knows, that he found, not in the substance of the brain only, but in every other tissue,—nay, even in inorganic substances,—solid fibres wound together in a serpentine manner. He, however, afterwards saw his error.

Fontana's observations were much more comprehensive; and he appears to have made out the structure of the brain and nerves such as has recently been demonstrated; though, from not being determined or delineated with sufficient accuracy, his observations led to no results. Hence what he did see correctly was attributed more to optical illusion than to reality.

In recent times two opinions, the converse of each other, and both erroneous, seem to have prevailed. Barba and others maintained the old view of Della Torre and Prochaska of globules in a mucus; other investigators admitted no globules, but fibres as the basis of the cerebral substance. Some persons, again, held a middle opinion. Hence, in physiological writings, we meet with such expressions as *globules of the brain*, *fibres of the brain*, or *cerebral tissue*.

The opinion that the nervous substance consists of fibres has been chiefly founded on examinations made of brain hardened by spirits, or from the existence of the lines and striæ visible to the naked eye and low magnifying powers. The medulla of the nerves has been pretty generally considered as prolongations of the cerebral substance contained in the membranous cylindrical tubes formed by the neurilemma. Among those who held the middle opinion, that the nervous substance consists both of fibres and globules, were Sir Everard Home and Mr. Bauer. Their observations, though made under every advantageous circumstance, are of no value. They appear, indeed, to have seen the structure described by Ehrenberg under the name of varicose or jointed tubes, but to have taken it for venous branches with valves; and, in correspondence with the notion, they have erroneously represented it as branched. Regular rows of globules, such as they represented their fibres to consist of, are not found in nature. Ehrenberg, in his historical notice, has not mentioned Sir Everard Home's doings expressly; and we only allude to them here to show how little worthy they are of being adduced along with those of Ehrenberg, as has lately been done by a contemporary critic.

At page 219 of our fifth volume we gave a short notice of the views of the structure of the nervous substance promulgated by Professor Berres, of Vienna, soon after Ehrenberg announced his discovery. Professor Berres has recently reiterated those views, both by description and delineation, in the fifth part of the work, the title of which we have placed among the others at the head of this article. Although we have done so, we have not thought it necessary to introduce any of his views into our discussions, because they are too little consonant with what is really found in nature. By this we do not mean to say that Professor Berres has asserted anything which he has not seen: on the contrary. But his error is that he has put down everything that he has seen; and, trusting to magnifying power alone, he seems to have forgotten that it is necessary to analyze what one sees; to separate, dissect, display, and to trace under the microscope. "No doubt," Burdach remarks (p. 8), "he has really seen all that he has described and delineated, but has subjected it to a false explanation." Valentin (*Repertorium*, vol. i. p. 61,) says, in speaking of Berres' account of the nervous substance, that, supposing it possible he might have overlooked, in his previous examinations, the relations of things described by Berres, he examined every point of the subject anew, and satisfied himself that most of Berres' assertions are incorrect. The motto affixed to Professor Berres' work is "Non conscripsi ad narrandum sed ad probandum." This we have great pleasure in saying, from our own observation of Professor Berres' injected preparations, is very applicable to that part of his work which treats of the ultimate distribution of the vessels; but the converse would have been more applicable to what he has written on the nervous substance.

In our attempt to collate the different modes of viewing the intimate structure of the brain and nerves adopted by the authors whose works lie before us, and to deduce what may be considered as established facts, we shall distribute the subject into three heads: 1, the intimate structure of the brain and spinal marrow; 2, that of the nerves and ganglions; and, 3, the mode of termination of the nerves.

I. THE INTIMATE STRUCTURE OF THE BRAIN AND SPINAL MARROW.

A. *The gray substance of the brain.* This consists, according to Ehrenberg,* (p. 19,) "of a close and very fine vascular network." Besides this, he has also observed, "at the very periphery of the gray or cortical substance, a soft mass consisting of very fine granules, in which are deposited, here and there, larger grains in detached heaps or in strata. The larger grains are free, and consist of still smaller granules; the very fine smaller ones of the mass appear simple; and, wheresoever their smallness, softness, and transparency allow us to form an opinion on the subject, they seem joined together in rows by tender threads. In the vicinity of the white or medullary substance, *the fibrous character of the gray or cortical substance becomes more and more evident;* and, in the same proportion, the blood-vessels become somewhat larger and fewer in number."

B. *The White or Medullary Substance of the Brain.* "This," says Ehrenberg, (p. 20,) "presents still more distinctly a fibrous structure. The fibres are, in fact, evidently direct continuations, gradually becoming thicker, of the finer cortical fibres. . . . They are not simply cylindrical, but resemble rather rows of hollow beads, not touching each other, but separated by the intervention of short tubes, or they resemble exactly tubes presenting dilatations at intervals." The tubes under consideration are always straight, and for the most part run parallel to each other; sometimes they intercross. Ehrenberg says that, in his almost innumerable examinations, he has only four times observed a division of the tubes into branches, but he never saw an anastomosis between any. In the bundles of tubes near the base of the brain, as also in the medullary substance lining the ventricles, there are always found tubes thicker than the rest, in which an outer and an inner line, indicating the boundary of the tubular walls, may often be perceived quite distinctly, and consequently the width of the bore or caliber determined. "Hence," says Ehrenberg, (p. 20,) "we can no longer speak of fibres or filaments as composing the brain, but of *tubes or canals presenting dilatations at intervals;* or, in other words, *varicose or jointed tubes or canals.*"

In regard to the spinal marrow, Ehrenberg says (p. 23,) that the gray substance of it, which is known to be situated interiorly, and the white substance, which is exterior, have the same structure as the corresponding substances of the brain.

In short, Ehrenberg gives out in the work before us (p. 37,) as esta-

* In his researches, Ehrenberg always uses the compound microscope, but he has not generally employed the highest powers. All the observations which he details were made by powers magnifying about 350 to 360 diameters. Under these powers, also, he made his drawings. Higher powers he has only used to determine and confirm what he saw with the lower.

blished facts: That the substance of the brain consists of tubes dilated at regular intervals, and which he calls *varicose* or *jointed tubes:* these tubes lie parallel to each other or in bundles, are from 1-96 to 1-3000 of a line in diameter, and run from the surface towards the ventricles and base of the brain, becoming thicker as they converge: they pass into the spinal marrow, the greatest part of which they form: they are not held together by any cement or cellular tissue that can be discerned: and that the spinal marrow of man, and all the great divisions of the vertebrata, consists of varicose or jointed tubes, exactly like the brain; with this difference, that the finer tubes (of the gray substance) lie inside, the thicker ones (of the white substance) outside, and all have a predominating parallelism one to another.

These results of Ehrenberg's researches into the structure of the brain, as well as of those into the structure of the nerves, by and bye to be noticed, were read before the Academy of Sciences of Berlin, in 1833, and an abstract of them published in Poggendorff's Annals of Physics for the same year. It was not long before they were put to the test by other observers; some of whom agreed with Ehrenberg, while others raised objections to certain of his views, and that on grounds more or less substantial. All admitted the general results; and, indeed, it could not be otherwise, seeing that they are not very difficult of demonstration.

Of those who have written on the subject, and offered corrections of Ehrenberg's views, since the latter first published his discovery,* the late distinguished physiologist, Gottfried-Reinhold Treviranus, of Bremen, Valentin (now Professor of Anatomy in Berne,) and Burdach, the younger, of Königsberg, deserve to be particularly named.

Treviranus (2tes Heft, p. 28,) distinguished in the gray substance of the brain fine cylinders, closely arranged and interwoven, which he calls *primitive cylinders;* and in the white substance what he calls *medullary cylinders*, each of which derives its origin from the union of several of the primitive cylinders. (p. 40.)

The primitive cylinders and the medullary cylinders of Treviranus are respectively the same things as the very fine varicose or jointed tubes described by Ehrenberg in the gray substance, and the larger varicose or jointed tubes, which, according to him, compose the white substance. So far there is little difference of opinion; but it will be seen that, according to Treviranus, several of the primitive cylinders unite to form a medullary cylinder; whereas Ehrenberg describes the tubes of the gray substance and those of the white as simple direct continuations of each other. The most palpable point in which Treviranus (p. 31,) differs from Ehrenberg is this: that he denies the beaded, varicose, or jointed form to be a constant and essential property of the tubes composing the substance of the brain, but declares it to be merely accidental; the simple effect of putrefaction, pressure, or other reagency after death. This assertion of Treviranus is now recognized to be correct, we believe, even by Ehrenberg himself. But it is to be kept in mind that the readiness with which the tubules composing the cerebral substance assume the

* Valentin mentions in a paper (in Müller's Archives, 1834, p. 409,) on the primitive fibres of the brain, that, several years before Ehrenberg published, Purkinje used to demonstrate them in his lectures. Valentin says that he himself saw them so demonstrated by Purkinje, in the session 1829-1830.

beaded, varicose, or jointed form is a remarkable and distinguishing characteristic.

Valentin (p. 93) considers the beaded, varicose, or jointed appearance as accidental; but, for distinction's sake, he still retains the name of *varicose fibres*, for which, as above seen, Treviranus employs the designation of *medullary cylinders;* a term which we had better not adopt, as it might be liable to be confounded with the cylindrical tubules of the nerves, which contain what Ehrenberg calls *medulla*, hereafter to be noticed. Valentin describes the gray substance as consisting "of an aggregation of globular masses, between which he finds in the yellow substance,—that is, the transition substance from the gray to the white, the numerous but isolated loop-like terminations of the cylinders of the white mass." We shall consider the globules of the gray substance when speaking of the ganglions.

The view given by Ehrenberg as to the course of the tubules of the brain from the surface towards the ventricles and base does not coincide with that of Valentin, who says (p. 92) he has satisfied himself "that the appearance to the naked eye, as if the individual fibres, or bundles of fibres, diverged, is quite unfounded. These bundles of primitive fibres form rather, according to him, the most beautiful and intricate plexuses, which present peculiar characteristics in different places." In regard to this assertion of Valentin's, Burdach, in mentioning that Valentin uses a curved scissors for cutting off pieces of the brain for examination, asks (p. 17) that, as this means is apt, by the pressure necessarily produced, to bring the component fibres into confusion, "might not, perhaps, the plexusf-ormations observed by Valentin in the substance of the brain and spinal marrow, be considered, in part, at least, as depending on the employment of the scissors; seeing that the fibres of a nervous bundle, which certainly run in a parallel direction, may, by pressure and shoving, be so easily changed into a plexus?" At page 24, Burdach again says, in regard to this, "Such manifold plexus-formations, or loop-like terminations of the cerebral fibres, as Valentin has observed and described them, I have indeed never seen; but consider the investigation of the course of the organic elements of the brain so difficult, that I by no means offer my observations, which were merely incidental, in opposition to those of Valentin."

Leaving out of view the differences of detail in the descriptions of the cerebral substance given by Ehrenberg, Treviranus, and Valentin, and the different modes of expression employed, we perceive that, taking them generally, they all agree in this, that the white substance of the brain is composed of fibres or tubes; and that the gray substance also is composed of fibres or tubes, together with granules. Excepting, then, that the varicose or jointed appearance of the tubes is not original and essential, and, even when they exist, perhaps not so regular as Ehrenberg represents them, but that they occur in consequence of post-mortem changes, and with greater or less irregularity, we may remark that the description, as given by Ehrenberg, agrees perfectly with what can be always readily demonstrated. Valentin's loop-like terminations of the cerebral fibres in the gray substance are not of such easy demonstration. As to the union of the fine fibres of the gray substance to form the thicker fibres of the white, as asserted by Treviranus, we think it more doubtful.

It is commonly considered a very difficult matter to demonstrate the primitive fibres or tubes of the brain, and that it is especially necessary to employ for the purpose very high magnifying powers. We can assert, from our own experience, that there is nothing more unfounded. A simple microscope, with lenses magnifying 150 to 200 diameters, will be found to serve the purpose very well. If then, to begin with, the observer will take a minute piece of recent brain at the root of one of the nerves, (say the auditory,) and spread it out carefully in a drop of water on the object-glass, he will not fail to see hundreds of tubes, presenting less or more the varicose or jointed appearance described by Ehrenberg, according to the freshness and undisturbed state of the brain, or contrariwise. Having thus once seen and become acquainted with the appearances, it will be easy to display and examine the tubular structure in all other parts of the brain. (See Fig. 1.) It is, of course, necessary to employ only very minute pieces of the thinnest possible slices of brain; and these will often require to be torn, separated, and displayed by means of needles.*

c. *Organization of the Primitive Fibres or Tubules of the Brain.* The interior of the so-called varicose or jointed tubules is throughout as clear as water: hence, says Ehrenberg, (p. 21,) they must contain some such matter as vapour, water, or clear jelly. Ehrenberg, however, was not able, even with a magnifying power of 3000 diameters, to determine exactly the nature of the contents: at least, he could see nothing of a granulary, or of any other particular appearance; and he is inclined to suppose that the contents are a perfectly transparent and tenacious substance. From our own observations, we can affirm that it is so. We have, in fact, seen it oozing out of the broken extremities of the tubules, and glueing them to the object-glass, so that they adhered like leeches; and the free ends of the tubules moved about when the water under which they were examined was agitated. A microscopical doublet, magnifying 150 to 200 diameters, will be found sufficient to show this, much better, indeed, than a higher compound power; because it is evident that to see a transparent fluid, it is not so much high magnifying power as good light and definition that are required. We shall hereafter see, as Ehrenberg says, that this very transparent and tenacious substance differs considerably in respect to tenuity from that contained in the cylindrical tubules of the nerves.

For the contents of the tubules of the brain, Ehrenberg (p. 22) proposes the name of nervous fluid, *liquor nerveus*, whilst he retains the name of nervous medulla, *medulla nervea*, for the substance contained in the cylindrical tubules of the nerves; and he suggests that the term medullary substance of the brain, *medulla cerebri*, if still retained, should be employed only as a conventional term. He thinks, however, that *white substance of the brain* would be better. We have already

* The structure of parts, such as it can be seen by the naked eye, now no longer suffices for physiology; we therefore counsel such of our young friends who would cultivate properly this most important branch of medical science to procure forthwith a microscope, and examine for themselves. It is not necessary to get an expensive instrument. Such a *simple microscope* as may be had of Mr. Ross, optician, in Regent street, Piccadilly, London, for six guineas, will enable any one to make himself acquainted with the intimate structure of every part of the body as far as it is yet known.

seen that Ehrenberg's so-called varicose or jointed tubules are called by Treviranus medullary cylinders, (Markcylinder.)

In regard to the nature of the walls of the tubules of the brain, Burdach supposes (p. 29) "that the elementary fibres of the brain and spinal marrow are not in general provided with sheaths of cellular tissue, but lie with their own substance in close contact, without the intervention of any other matter. In this case they should consist of a substance, the outer peripheral part of which, being more viscid and of greater consistence, would form a sort of rind or shell; whilst the inner, central part remained fluid. Our own observations lead us to agree with Burdach. The following extract from Wharton Jones, describing the investment of the newt's ovum, illustrates very well what Burdach means: "The ovum of the newt differs from that of the frog, inasmuch as the gelatinous-like matter which surrounds the yelk and its membrane is of an oval form, *and is somewhat hardened on the surface so as to form a kind of shell, inside which is a fluid substance*, in which the yelk and its membrane can freely revolve and glide from one end to the other.*

### II. INTIMATE STRUCTURE OF THE NERVES AND GANGLIONS.

A. *Cerebro-spinal Nerves and Ganglions.* According to Ehrenberg, (p. 24,) "all the nerves examined by him, with the exception of the optic, auditory, and olfactory nerves, and the sympathetic in the middle of its course, consist of cylindrical tubules, of an average thickness of 1-120th of a line,† running parallel to each other, and never anastomosing." The latter fact, indicated by Fontana, and afterwards by Prevost and Dumas, was first demonstrated by J. Müller, who showed that the primitive fibres of the nerves run in uninterrupted continuity from the centre to the periphery, always lying merely alongside each other, and nowhere dividing or inosculating. Kronenberg (*Plexuum Nervorum Structura et Virtutes,* Berolini, 1836, 8,) gives, by his researches, additional confirmation to this opinion.

The cylindrical tubules, united together in bundles, form the nervous cords. Each single bundle, and the whole cord, are surrounded by a vascular fibrous sheath, called *neurilemma*. In reference to the neurilemma, we would here introduce the following observations by Burdach, who, we may premise, has principally occupied himself with the investigation of the structure of the spinal nerves; having directed his attention only incidentally to the study of the substance of the brain and spinal marrow, and that merely for the sake of comparison with the substance of the nerves. The nerves he examined were for the most part those of frogs, and sometimes also of the smaller fishes, birds, and mammifera; as (pp. 9, 10,) "nerves just removed from the living animal are alone fit for the examinations to be instituted." According to Burdach, (p. 18,) when we examine a nerve in the undisturbed state, an undulated appearance is observed in it lengthways. This is owing, *not*, as Valentin supposes, to a contracted state of the neurilemma,—to an alternate elevation

* On the First Changes in the Ova of the Mammifera, &c. (In Phil. Trans. Part ii. for 1837, p. 339.)

† The cylindrical tubes, Ehrenberg says, are found, both thickest and thinnest, in the invertebrata; being sometimes as thick as 1-48th of a line, and in other cases as thin as 1-1000th of a line in diameter.

and depression of the fibres of the cellular tissue constituting the neurilemma; just, in fact, like the wrinkled state of the investment given to the cords of spiral wire in elastic garters; *but*, according to Burdach, to the primitive fibres of the nerves which lie in an undulated position, the neurilemma being shorter than they. "We have here," says Burdach, (p. 19,) "a wise arrangement of nature, by which, on the occurrence of any pulling and tearing of a part provided with nerves, the neurilemma must first be considerably stretched before the extension can affect the primitive fibres, which are relatively longer, and lie loose in their investment." According to Valentin's view of the matter, on the occurrence of any pulling or tearing, the primitive fibres would be the first to suffer, in the same way that the coil of wire in elastic garters is drawn out considerably before its sheath, being stretched to the full, can prevent further extension or elongation.

To return to the cylindrical tubules of the nerves. Ehrenberg says he has satisfied himself that the individual tubules are not invested by any covering of neurilemma. Very frequently the different nervous bundles of one and the same nerve unite by what Ehrenberg calls a false anastomoses: that is, the tubules of one bundle enter into and pursue their course in another, without, however, as has been already pointed out, the individual tubules ever inosculating.

It is a fact established by the observations of Ehrenberg, Treviranus, and others,—and to the correctness of which we ourselves, from our own observations, subscribe,—that the cylindrical tubules of the nerves are direct continuations of those of the brain and spinal marrow. The latter become thicker in caliber, and stronger in their walls in the transition. This transition part of the nervous tubes undergoes less readily the change to the varicose form; some slight dilatations only presenting themselves.

According to Valentin, the primitive fibres of the spinal nerves do not terminate in the spinal cord, but pass on to the brain. The primitive fibres of the nerves which join the extremity of the spinal cord run forward; whilst those of the nerves, entering the spinal cord laterally at its upper part, proceed first transversely towards the interior of the cord, as far, or nearly as far, as the gray substance, and then follow the same longitudinal course to the brain as the others. In the white substance the fibres lie side by side; but, where the white and gray substances touch each other, the fibres are separated by the intervention of the globules of the gray matter, presently to be described, and at last radiate through the cortical substance, where (as we have already mentioned), Valentin says, they form loop-like terminations, by uniting with one another. This is most distinctly seen at the point of union of the white and reddish-gray substances, or in the yellow substance at the periphery of the hemispheres of the cerebrum and cerebellum. (For what we have to say of the ganglions of the cerebro-spinal nerves, see under the head "Sympathetic Nerves and Ganglions.")

B. *Organization of the primitive Fibres or Tubules of the Nerves.* "The purely cylindrical tubules of the nerves," says Ehrenberg, (p. 25,) "differ from the varicose ones of the brain essentially in this, that they have stronger coats, a much larger caliber, and contain in their interior a very evident and less transparent medullary substance, which appears to have been often observed before, though not very accurately." This medullary substance, much grosser than that contained in the tubes of the cerebral substance, can be readily seen oozing out of the cut extremity of the nervous tubule, and diffuses itself in the water under the form of irregular shaped flakes. (See fig. 2.) Remak (*Müller's Physiology*, and also his *Archiv.* 1837, p. iv. *Jahresbericht*,) describes the contents of the nervous cylinder, as Fontana had done before, to be either a perfectly solid fibre, of rather less diameter than the cylinder itself, or a pale flat filament, separable by pressure for a considerable extent here and there from the investing tube, which is prone to become wrinkled or puckered. He could not detect any more minute fibrous structure in this filament, although it sometimes becomes split into several threads. Remak further states, that the contents of the cerebral tube is, as in the fibres of the nerves, a coherent thread; but, like the tube itself, of a much more delicate nature. The simple expression of all this is; as we have already stated,—the contents of the cerebral and nervous tubules is a viscid substance, which, allowed to ooze out slowly, coagulates into an irregular shaped flake; but, if drawn out, whether by pressure or any other means, it is spun into a thread.

As to this subject of the contents of the primitive fibres of the nerves, "I have," says Burdach, (p. 21,) "never been so fortunate, even by examination as soon after death as possible, to find the primitive fibres of a bundle wholly and throughout with perfectly clear contents: there was always in them, here and there, a mass consisting of irregular round particles, which probably, for the most part, gives, by the refraction of the light, a darker appearance to the primitive fibres." The contents Burdach observed to ooze out in the form of a clear, thick, fluid, colourless mass; which was some time after distinctly converted again into a mass of irregular globular particles. Ehrenberg thought that the contents of the cylindrical tubules of the nerves was a granulous fluid; but we agree with Valentin (p. 115) and Burdach, that the substance contained in the primitive fibres of nervous substance in general, both of the brain and of the nerves, is a half-fluid, somewhat viscid, transparent, oily-like matter, which is changed into a granulous mass only by coagulation. Treviranus (p. 38) describes the contents of the nerve-cylinders as a soft matter, in which globules are sometimes seen.

Ehrenberg says, (p. 26,) that he has convinced himself in many ways that the fibres of the nerves are hollow. "In the first place, there are distinctly perceptible in each tube four parallel lines, two of which form the outermost boundary lines, but the inner two indicate the limits of the internal cavity. In the second place, a view of the tubes filled with medulla is very easily obtained by spreading them out with two needles, so as to avoid causing any or much pressure. By now laying a plate of glass over the part, and pressing it slightly, the tubes, which were previously filled with medulla, are seen quite empty, and the medulla forms at their ends a thick protuberance. In the third place," he continues, "I have been able to perceive, during the observation, even the movement of the contained mass caused by gradual and gentle pressure; and, in the fourth place, I have often, in transverse sections, perceived the *lumina* of the individual tubes most distinctly." "Each of these reasons," he adds, "and, much more, all taken together, indisputably prove the fact that the fibres of the nerves are hollow." The transition of the tube of the nerves into the primitive fibres of the brain Ehrenberg adduces as a proof of the tubular nature of the latter. He says, (p. 25,) "All doubt on this point is removed by the fact that the cylindrical tubes of the nerves pass directly into the varicose or jointed ones of the brain."

As to the inner boundary lines of the primitive fibres of the nerves admitted by Ehrenberg, Burdach looks upon them as an effect of the refraction of the light, produced by the fluid contents of the primitive fibres being more accumulated at the edges, causing this part to project above the centre. Burdach enters very much at large into the intimate organization of the primitive fibres of the nervous substance. We shall not follow him throughout all his enquiries; the following only we give. The way he accounts for the production of the varicose appearance which the primitive fibres assume is, (p. 29,) "That the contents of the primitive fibres of the brain and nerves undergo throughout a peculiar tendency to assume the state of globules after the extinction of life, but while they are still fresh; that the sheath of cellular tissue opposes this tendency more or less successfully, according to its strength, and the more so as the contents adhere in some manner to its inner surface by virtue of their viscidity. Hence varicosities are produced in the strong-sheathed primitive fibres of the peripheral nerves only after the action of mechanical or chemical influences, and that but imperfectly. In the same fibres, on the contrary, when, by reason of youth, they are thin-walled, the varicosities are more regular. Further, in the more or less tender fibres of the cerebral nerves, they appear with different degrees of distinctness and constancy; lastly, in the extremely delicate-walled fibres of the brain and spinal marrow, the varicose appearance occurs in the most regular and constant manner."

In regard to the question whether there be any movement or circulation of the contents of the tubules of the nervous substance, Ehrenberg says, (p. 27,) "My investigations hitherto on the nerves of living animals have not yet perfectly shown to me any circulation; and Leeuwenhoek likewise denies it distinctly. But whether Leeuwenhoek, at the place where he speaks of movements seen in the canals of the optic nerve of the eye of the ox, observed a circulation, however indistinct, is uncertain. This subject, as one of the highest importance to physiology, and one the decision of which is within our reach, I recommend to scientific investigators for their cooperation; particularly as, according to my experience, it is not very easy to reduce it to demonstration. Mere hasty assertions, for or against it, deserve no notice." Emmert (p. 30) lays down the following proposition: "The phenomena of muscular activity and loop-like mode of termination of the fibres of the motor nerves render it likely that there is a continuous current; a circulation of nervous fluid in the motor nerves." This proposition Emmert illustrates at considerable length, but does not support by very close reasoning. Burdach's experiments of tying a nerve (p. 42) do not favour the idea that there is any movement of the medulla in the primitive fibres; any real current of it in a particular direction.

Are the fibres or tubules above described really the ultimate elements of the nerves? Müller doubts that they are so, when he considers the great size of the so-called primitive fibres of the nerves as compared with the minute elementary parts of the muscles, the cellular and other tissues. Treviranus appears to think that the cylindrical fibres of the nerves are composed of more minute elementary fibres closely arranged alongside of each other: this he infers from having remarked stripes in the cylindrical tubes, running lengthways. In addition to this, Müller mentions that Schwann saw in the mesentery of the frog nervous fibres, of the thickness of the primitive fibres, from which still finer fibres ran out. The question resolves itself into this: Does the half-fluid contents of the nervous fibres or tubes, above described as primitive, present, in fact, an appearance not homogeneous, but as if composed of minute threads or filaments of half-fluid consistence? Neither Valentin, Burdach, nor we ourselves, have been able to see any such thing in the course of the nerves. We shall recur to this subject when speaking of the terminations of nerves.

c. *Is there any difference of Structure between the primitive Fibres of Nerves of Motion and of common Sensation?*—In the roots of most nerves, where they emerge from the surface of the brain and spinal marrow, Ehrenberg (p. 24) observed among the cylindrical tubes so-called varicose ones, nearly as thick, but which were for the most part likewise filled with distinctly visible nervous medulla. "Whether these mixed nerves, as they may be called," asks Ehrenberg, (p. 25,) "be the sentient, and the purely cylindrical ones be the motor, is a very important subject for further investigation?" In the supplement to his memoir, (p. 43,) Ehrenberg informs us that he examined, with Professor Müller, the sensiferous and motiferous roots of the nerves in the frog, and found no important difference in the microscopical structure. Lauth, and after him Remak, discovered so-called varicose or jointed tubes in different nerves. Remak says (p. 146) he found, in the sciatic nerve of a frog, where it divides into the peroneal and tibial nerves, *a large mass of varicose fibres.* He afterwards convinced himself that there are no spinal nerves without varicose or jointed fibres. All this, however, does not appear to be connected with a sensiferous or motiferous function, but to be merely a stage of development; for the occurrence of so-called varicose or jointed tubes is quite common in young animals; perhaps, simply for the reason we have above mentioned, viz. that the walls of the nervous tubules, being more delicate, they more readily assume the varicose form. Thus, according to Remak, (p. 148,) in a young rabbit, the second day after birth, all the cerebro-spinal nerves, throughout their entire course, consist of *transparent varicose fibres:* in other words, the primitive fibres or tubules of the nerves, being very delicate, like the fibres of the brain, readily assume, after death, the varicose appearance. In young but not very small frogs, Burdach (p. 38) frequently found in the ischiatic nerve, in addition to the cylindrical tubules, still some which had the varicose appearance; whilst, in a completely grown frog, it was always in vain that he looked for varicose tubes. In regard to the *roots of the spinal nerves,* Burdach (p. 10) could only confirm Ehrenberg's statement that both the sentient and motor roots exhibited no actual difference in regard to the appearance of the primitive fibres, except that those of the posterior roots appeared somewhat thinner; a remark which coincides with that already made by Emmert, (p. 9.) Burdach could find no fibres readily assuming the varicose form in the ischiatic nerve of the full-grown frog, even at its division into tibial and peroneal, as Remak had done. He found no so-called varicose tubes in the cutaneous nerves of the frog; only proportionally finer cylindrical tubes. Burdach therefore comes to the fixed conclusion that, in the spinal nerves of the adult frog, in the fresh state, and quite unchanged by compression or any other kind of treatment, there are no varicose fibres. He thinks all that can be inferred from Remak's researches is, that the elementary particles of the nerves acquire their full, regular development only after the complete and perfect growth of the individual; that, on the contrary, at an early period, they are, both in regard to their contents and their investments, more amorphous and more tender, and by handling easily assume an appearance not natural to them. "Hence," says Burdach, (p. 10,) "I have found young frogs, new-born rabbits, and recent human embryoes, absolutely useless for my investigations."

In regard to Remak's researches, it is to be remembered, however, that he distinctly says, in speaking of the greater thickness of the muscular than of the sentient nervous fibres, "These differences are so striking, that, putting out of view altogether the kind of fibres (varicose and such like), we can determine, merely from their thickness under the microscope, whether we have before us a muscular or a cutaneous nerve." Of the three nerves of the tongue, the lingual, according to Remak, resembled a cutaneous nerve; the glosso-pharyngeal contained at most places regular, fine varicose fibres, so that it differed from a mere cutaneous nerve; and the hypo-glossal was quite as a muscular nerve. The above remarks refer to the nerves of rabbits four or five weeks old. In full-grown rabbits, Remak found the varicose fibres,—that is, fibres capable of assuming the varicose appearance,—very rare; and the transition fibres,—that is, fibres somewhat less prone to assume the varicose appearance,—less numerous than in the earlier period. The difference between cutaneous and muscular nerves he found as striking as before; but it consisted, for the most part, merely in the thickness of the fibres, and the greater quantity of fibres without medulla (?) found in the cutaneous nerves; whilst the varicose fibres do not now any longer so generally occur, or are so characteristically frequent in the cutaneous nerves, and much less so in the muscular. Of the three nerves of the tongue, the hypo-glossal continued to present the characters of a muscular nerve, the lingual of a cutaneous nerve, and the glosso-pharyngeal contained the most varicose fibres and cylindrical fibres without medulla.

It thus appears that, with the exception perhaps of a slight difference in thickness, the primitive fibres of sentient and motor nerves have exactly the same appearance under the microscope.

We have already mentioned that we thought Burdach had done well in describing the appearances which the nervous substance presents under different influences, at different periods of life, after different kinds of death, and at different periods after death, as well as in the morbid state. Burdach's work contains intrinsic marks of the observations and experiments having been most carefully made and faithfully reported. Whatever conclusions, therefore, he draws from his researches are entitled to our best attention. We have already noticed some of the most important points of his observations on the structure of the nerves: we shall not follow him more particularly, but simply content ourselves by extracting his own summary of his researches.

"1. The tendinous appearance visible on the surface of whole nerves, or thick bundles of nerves, does not depend on undulating bendings of the cellular tissue forming the sheath, but on a serpentine position of the bundles of the primitive fibres within the sheath.

"2. The nerve appears to retain its sheath even within an organ.

"3. The primitive fibres are not finer within an organ than they are outside one.

"4. The contents of all primitive nervous fibres are, in the natural state, a clear, thick fluid, which is only changed by coagulation into a granulary mass.

"5. The primitive fibres are at first cylindrical, but sink in the middle after death, and when they are laid on a plane surface; whence, by the refraction of the light, they present the appearance of double boundary lines.

"6. The knotted or varicose form is, indeed, peculiar to the primitive fibres of the brain and spinal marrow, but not essential; and depends simply on this, that the contained medullary matter possesses a tendency to assume the globular form, and thereby to overcome the resistance of the sheaths.

"7. There are many circumstances and appearances which render it probable that the primitive fibres of the brain have not a sheath of cellular tissue, but consist of a somewhat more viscid cortical and a somewhat more fluid central substance.

"8. Cold contracts, heat expands, the nervous fibres. Water is indifferent. Vinegar dissolves and softens, first the cellular sheath, then the medulla: potass first the medulla, and then the sheath. Spirits of wine coagulate the medulla and thicken the sheath. Creosote and corrosive sublimate exert a similar action. Alum and saltpetre dissolve both sheath and medulla: common salt less so; and prussic acid appears to render fluid and expand the contents of the primitive fibres.

"9. The nervous fibres attain their full development more slowly than other organic structures: they consist originally of granulous masses, and pass through the varicose* form gradually, but not quite equably, to the cylindrical form.

"10. By age it is only the general nervous sheath and the neurilemma that become thickened; the primitive fibres themselves are not visibly changed.

"11. Decomposition by putrefaction goes on most quickly in the brain and spinal marrow, less so in the nerves of sense, most slowly in the peripheral nerves. Putrefaction takes place in the brain and spinal marrow more quickly when left in the dead body; in the peripheral nerves, more quickly when they are removed, and laid in water.

"12. After hemorrhage, the primitive nervous fibres have a torn collapsed appearance. After death by suffocation, the peripheral nerves are also gorged with blood. After death produced by prussic acid, the fibres of the brain appear in a cylindrical form, and quickly fall down into clear globules.

"13. The blood-vessels going to the nerves *do not penetrate betwixt the primitive fibres, but merely twist round the bundles of fibres, in a net-like manner.*

"14. In dropsy, and perhaps also by inflammation, (dropsy and inflammation in frogs, caused by cauterizing the skin with lunar caustic,) the primitive fibres of the nerves acquire the appearance of transparent tubes filled to bursting with fluid.

"15. Nerves which have been cut do not reunite *immediately*, but only by the *intervention* of cellular tissue: they appear closed at their cut end by medullary matter which has oozed out. No new nervous twigs enter into the cicatrice of healed wounds.

"16. In the primitive fibres of the nerves, there is no movement of the medullary mass in a particular direction." (p. 43.)

D. *Structure of the primitive Fibres of the Nerves of the three higher Senses.* We have seen that Ehrenberg considers the structure of the ol-

* It would be more correct to say "that state in which the varicose appearance is so apt to take place."—Rev.

factory, optic, and auditory nerves as different from that of others. He describes them as composed entirely of the so-called varicose or jointed cerebral tubules. On this subject Valentin says (p. 52) the olfactory nerve has, almost along its whole course within the skull, an extremely delicate, fine, parallel, fibrous structure; the elements of which, lying close beside each other, constitute varicose threads. The optic nerve, on the other hand, is divisible into a multitude of thin bundles lying alongside each other, and visible to the naked eye; which bundles consist of a sheath of cellular tissue, and the fine primitive nervous fibres therein contained. The auditory nerve is distinguished by the peculiar fineness of the nervous fibres. According to Treviranus, (p. 36,) "The olfactory nerve consists, at its entrance into the nasal cavity, of bundles of cortical cylinders, uninclosed in any sheath; the optic nerve of medullary cylinders resembling those of the medullary substance of the brain; the auditory nerve of cylinders like those of the muscular nerves, but thinner." Volkmann could not always find varicose fibres in the nerves of special sense.*

The above views we consider in some respects right, and in others wrong. According to our observations, the olfactory nerves within the cranium consist of small tubules, similar to those of the substance of brain; but the fibres of the olfactory nerve, where it ramifies in the pituitary membrane, present the thick-walled, cylindrical, tubular structure, though very fine, of other nerves. According to the observations of Wharton Jones, the auditory nerve, from its origin to where it enters the internal auditory meatus, presents most distinctly the delicate-walled, tubular structure of brain; but, within the meatus, it assumes the ordinary thick-walled, cylindrical, tubular structure of nerve. The same physiologist says further, "Examined under the microscope, the cylindrical tubules of the cochlear nerve appeared to me to be larger than those of the vestibular, and to contain, or at least to give out, a greater quantity of nervous medulla."† In regard to the optic nerve, it is composed throughout of the so-called varicose or jointed tubules, or the medullary cylinders of Treviranus, bound together by sheaths of cellular tissue, in bundles visible to the naked eye. The optic nerve thus appears to differ from the olfactory and auditory nerves, and indeed from all other nerves, in nowhere presenting the thick-walled tubular structure: we think, however, that even in it we have observed a slight approach to such a structure, at that place where the nerve becomes somewhat constricted, just before entering the eyeball. Müller says he has found the fibres of the optic nerve in the sheep, when recent, much more minute than they appeared when examined later. We have observed the same thing, we may say, of the cerebral tubes generally.

E. *Sympathetic Nerve and Ganglions.* According to Ehrenberg, (p. 38-40,) the sympathetic nerve has a mixed substance, consisting of so-called varicose or jointed tubes and cylindrical tubes. Valentin says (p. 85) of the sympathetic nerve, that its primitive fibres, examined under the *compressorium* in an uninjured ganglion, exhibit perfectly straight boundary lines, like the peripheral nerves in general. Müller says, "It

* Neue Beiträge zur Physiologie des Gesichtssinns.—Leipz. 1836.

† See article "Organ of Hearing," in vol. ii. of the Cyclopædia of Anatomy and Physiology, p. 540.

is well known that the fasciculi of nervous fibres in the sympathetic nerve have, for the most part, a gray aspect, while those of the cerebro-spinal nerves are white: but the latter nerves also contain some few gray fasciculi mingled with the white and in many parts of the sympathetic nerve there are white fibres mingled with the proper gray or organic fibres." The sympathetic nerve having connexions with the anterior and posterior roots of the spinal nerves, the white fibres are derived from that source, and are both motor and sentient. It is a question, however, whether the sympathetic really contains any fibres *sui generis*. Valentin does not think so: he says, "the gray appearance of the cords of the sympathetic does not depend on any peculiarity of the fibres, but on ganglionic globules distributed here and there." Müller is inclined to think that the gray fibres of the sympathetic, after their connexion with the ganglion, are functionally different from the motor and sentient white fibres, which the latter derives from the cerebro-spinal nerves; yet, says he, it would be more simple and agreeable to suppose that all fibres, without exception, were the same, and differed only by the direction of the current or oscillation which took place in them. "However," he adds, "we are not come to that point of theory, and it still remains a remarkable fact that twigs of the sympathetic mingle themselves in other nerves, and still retain their peculiarities."

Of the *ganglions*, Ehrenberg says, (p. 31,) that they differ in structure. Almost all have this in common,—that they consist of collections of varicose cerebral tubules, which either alone form the ganglion, as is the case with the *chiasma opticum*; or they are mixed with thick cylindrical nervous tubules, as was found in all the ganglions of the sympathetic examined by Ehrenberg. In the case of the latter, the nervous tubules are enclosed in a delicate close vascular network, in the meshes of which are granules, such as are found in the retina and the cortical substance of the brain. In following the course of the varicose tubules of the ganglions, Ehrenberg could perceive very distinctly that they gradually became thicker until they nearly equalled the cylindrical nervous tubes in thickness; still they always retained their peculiar varicose structure. "The idea," says Ehrenberg, "that ganglions may be compared to small brains, is favoured by a knowledge of the structure." Thus, "the substance of the ganglions consists of a mixture of vessels and very delicate, scarcely distinguishable varicose tubes; in fact, of true cortical substance, and of a preponderating quantity of thicker varicose tubes; in fact, of true medullary substance. This cerebral substance lies around cylindrical nervous tubes, which do not become changed in it, but are strengthened by an admixture of varicose tubes in their bundles."

According to Valentin, (p. 78,) the original type of the ganglionic structure consists in this, that one or several bundles of fibres, which enter into the ganglion, form within it a more or less intricate plexus, according to the nature and size of the ganglion. But besides this, individual primitive fibres, or isolated bundles, consisting of very few fibres, run round on all sides of the peculiar ganglionic globules (see fig. 3). These globules are composed of an external fine coat of cellular tissue, within which is a nucleus, in the circumference of which is a second smaller nucleus; they have often also pigmentous deposits on them. These ganglionic globules may be very readily demonstrated in the *locus*

*niger*, and other black spots of the brain. The gray substance of the brain and spinal cord is formed wholly of the same globules as the ganglions of the vertebrate animals. The appearance of minute granules is produced by the disintegration of the original globules, which are very soft. The only circumstance in which the globules of the gray substance of the brain differ from those of the ganglions is, that the cellular tissue which invests the former is more delicate. In the white substance of the brain there are no globules or granules; any appearance of granules in it is produced by the nervous fibres being broken up. On the quantity of the deposition of gray globules between the primitive fibres depends the degree in which certain parts of the brain differ in colour from the white or fibrous substance. Where the primitive fibres are in greatest number, the colour is whitish gray; where they are less abundant, it is reddish gray; where, on the contrary, there exist only simple terminating loops of fibres between the globules, the mass is yellow. The darker colour of certain portions of the brain depends on a pigment deposited on the globules.

Farther, Valentin describes the structure of the ganglions of the cerebro-spinal nerves as not differing essentially from that of the ganglions of the sympathetic; but in them the pencil of fibres is more distinctly seen passing through unchanged between the globules of the proper substance of the ganglion.

Somewhat resembling the ganglionic globules above described, Ehrenberg observed, in the interior of the ganglions of the leech, large club-shaped bodies, filled with granules, and presenting a clear nucleus in the centre. Ehrenberg had frequently seen similar bodies in other parts of the nervous substance, but their connexion always remained indistinct to him. He describes the club-shaped bodies in the leech as forming eight bundles, two of which are prolonged into each of the four arms of the ganglion by means of long cylindrical tubes. Valentin has described similar bodies as existing in the ganglions of the abdominal cord of the leech. He saw globules which, like those of the ganglions of the higher animals, contained a nucleus. In this nucleus there was, at a point close to the surface, a reddish body, and sometimes, instead of this, there were several smaller corpuscles. Purkinje saw similar caudate bodies in the yellow mass between the cortical and medullary substance of the hemispheres of the cerebellum. He describes them as having a bright nucleus in the interior, and corresponding to it a smaller one on the surface. They are arranged side by side; their rounded extremities turned in towards the white substance, their tail-like prolongations out towards the cortical substance. As similar to the above, Müller regards certain club-shaped nucleated bodies, which he found in the medulla oblongata of the cyclostomatous fishes, (in a petromyzon preserved in spirits.) But they were in this case of a peculiar shape: their thick extremity was seldom rounded, but for the most part dentated; there being two, three, or four tooth-like processes, the form and position of which varied very much. Emmert (p. 8) mentions his having found, in the upper part of the spinal marrow of a rabbit, below the medulla oblongata, on the posterior surface of the spinal marrow, several club-shaped bodies which lay lengthwise in the spinal marrow beside and upon each other. "At one end," says Emmert, "they are clubbed and bounded by a curved line, at the other end they pass into fibres; their club-shaped swellings are not all of the same size, the line bounding them being sometimes more, sometimes less, distant from each other."*

III. TERMINATIONS OF THE NERVES.

A. *Terminations of the Nerves in the external Skin and Muscles.* Some years ago Rudolphi, in Germany, and Prevost and Dumas, in France, described what they supposed to be a termination of the nerves in the muscles by loops, but they appear to have referred only to loops formed by the larger nervous ramifications. This is especially the case with Rudolphi's observations which were made by the unassisted eye.

Valentin and Emmert discovered the mode of termination of the nerves in the muscles about the same time, but by a different procedure as regards the manipulation. Burdach confirms the accounts of Valentin and Emmert, and gives, in addition, the mode of termination of the nerves as he has made it out in the skin of the frog.

What we have hitherto extracted from Burdach's work is contained in the first section. Having only incidentally examined the structure of the brain and spinal marrow, he has limited himself chiefly to the investigation of the structure of the spinal nerves. "Although," says he, "these my investigations, so far as they have hitherto gone, have yielded by no means brilliant results, and are to be looked upon rather as a work preparatory for further researches, I have nevertheless been induced to communicate them in the first section of these sheets, seeing that I have been conducted by them to the observations contained in the other two sections; which might, indeed, appear more worthy of being made known."

We shall first consider the mode of termination of the nerves in the muscles, as that was first discovered, and as it is more simple than the mode of termination of the nerves in the skin.

In order to demonstrate the point, Emmert stretches an abdominal or pectoral muscle of a frog on a plate of glass; and, when it has adhered to the latter by drying slightly, he shaves it thin with a cataract knife. The following is his description of the mode of termination of the nerves in the muscles as demonstrated in this manner. "In what way soever the terminating nervous fibrils arise, they have always the same course and the same termination. They go off from the other primitive fibres, in the direction towards which the farther expansion of the nervous cords

* The nucleated structure of the globules of the gray substance of the brain and of the ganglions is not peculiar to those parts: it is found in the corpuscles of cartilage, in the epithelium of the mucous membranes, in the epidermis. Werneck, of Salzburg, has described a new layer within the capsule of the lens, (See our last Number, p. 207,) which, as we can testify from our own observations, consists of small bodies with nuclei in their middle, like the small bodies of the epithelium. The small bodies of the membrane of the pigment of the eye also present the central nucleus. We have found also nucleated bodies in the bulbs of the eyelashes. Valentin communicates observations by Purkinje, which he himself has repeated, on the structure of the choroid plexus; viz. that these shaggy organs are covered by a fine transparent epithelium, the globules of which have a polyhedral circumference. Each globule contains in its middle a dark round nucleus. In man, the middle of each cell has externally a round granule of pigment corresponding to the central point of the position of the nucleus in the inside. The vibratile cilia of various surfaces are connected with the corpuscles of their epithelium.—REV.

is carried, consequently, towards the periphery; they run in a serpentine manner over the muscular fibres; and, having described on them a larger or smaller arch, they return to another nervous cord or another nervous bundle, or unite with some other single primitive fibre which has just run a similar course, and accompany it to a nervous cord, to which they apply themselves and in which they then go backwards to the larger nervous stems. The arches of the primitive fibres which are formed in this manner on the muscular fibres, and of which the convexity is directed towards the peripheral expansion of the nerves, exhibit multiplied diversities in regard to the size and form of the curve." (p. 19.)

Valentin, in his examinations, made use of the recti muscles of the eye of man and the smaller mammifera, the cutaneous muscles of the mammifera, the abdominal muscles of different small animals, all the muscles spread on the inner surface of the cavity of the trunk, and, lastly, the nervus intercostalis internus of rabbits, guinea-pigs, &c. These he examined without any farther preparation than under the *compressorium.* Burdach makes use of a thin muscle, previously laid a few minutes in vinegar, which has the effect of rendering the muscular substance of a horny transparency. During the examination he employs slight compression. By this mode of examination, Burdach always found the termination of the nerves in the muscles as follows.

"To each individual muscle there goes, in general, only a single nervous stem. . . . The nerve, having entered the muscle, first runs downwards in it to some extent parallel with the muscular fibres . . . After this pretty straight course, the nerve begins to split into thicker and thinner branches, which again subdivide into twigs of a few primitive fibres. These branches and twigs proceed over and under the muscular fibres obliquely, or with a slight arch; more rarely quite across; frequently intercross, and then form, as they approach nearer the end of the muscle, a plexus, by frequently joining and separating from each other. This is what Valentin calls the *terminating plexus.* By means of it is produced a frequent interchange of primitive fibres among the twigs of the same branch, or of different branches, or even of different nervous stems, in the case where the muscle possesses several such. From this plexus there come forth, lastly, at a point still nearer the end of the muscle, ramuscules, which, being resolved into single primitive fibres, or bundles of very few fibres, form arches or loops, the convexity of which looks towards the end of the muscle, the concavity towards the stem of the nerve. Thus are formed Valentin's so-called *terminating loops.* After having formed the loops, the single primitive fibres, or bundles of a few fibres, again unite together, and re-enter the plexus, and through this return to their nervous stem." (p.54.) See fig. 4.

Burdach's examinations into the mode of termination of the nerves of the skin were made on the integument of the frog; and for this purpose he found very convenient a circumstance he discovered,—viz., that, by the action of vinegar, the skin of the frog may be divided into three layers.

The integument of the frog covers the animal quite loosely like a sack, and is only here and there connected with the subjacent parts, particularly where blood-vessels and nerves enter into it or pass out from it, and where cutaneous muscles are inserted. Of the three layers into which the skin is divisible, the outermost is the epidermis; the second, the corpus mucosum, or seat of the pigment; and the third, or innermost, is the chorion. This layer of the skin consists of dense cellular tissue. In it we find the cutaneous nerves, blood-vessels, and glandular structures lying spread out before us. With the exception of the part lying on the middle of the abdomen, this chorion, as long as it is in the moist state, is so transparent that, whether the microscope be directed to the inner or outer surface of it, the whole expansion of the nerves can be seen from the cut end of the trunk, lying as yet outside the skin, to the very finest ramifications, without a single twig escaping the eye.

"As soon as the trunk of the nerve enters the skin, it divides into three or four branches, which run in a diverging manner towards opposite sides. The cutaneous nerve first runs to some extent rather parallel with the skin; but after that must make a bend, however slight, in order to penetrate into the skin. . . . . Most frequently the division of the nerve is into three branches: where the trunk divides only into two, (a very rare circumstance,) one of the two, or even both, give off, after a short course, a thick twig which runs in an opposite direction. The branches which go off divergingly now ramify in their slightly serpentine course. . . . The twigs given off sometimes again join the parent branch, or some other branch of the same stem, after a very short course; but more frequently they maintain for a longer time their independent course, in which case they become thinner and thinner by dividing and by the giving off of ramuscules, consisting of more or fewer; sometimes even of only a single primitive fibre. The branches which have become thin by giving off twigs, the thickest as well as the most slender twigs, as well, lastly, as the bundles consisting of a greater or less number of primitive fibres, or even single primitive fibres, arising from these in the shape of ramuscules, form with each other and with the branches, twigs, and ramuscules* of other nervous stems, a very complicated plexus. In this they sometimes join each other by apposition; sometimes, again, separate from each other by dividing and ramifying. If we now follow a nervous branch continuously through this network, overlooking for the time all its twigs in their farther course, we find that when, in consequence of giving off ramifications, it has diminished to a few primitive fibres, it again gradually increases in thickness; seeing that new bundles, sometimes thinner, sometimes exceeding itself in thickness, always join it and accompany it. Being thus more and more enlarged, it appears at last as a branch of quite another nervous stem. The same thing is found to be the case in regard to every other, even the smallest of its twigs, and ramuscules. We can trace each of them to a foreign nervous stem, through the most complicated unions and separations of the network. From this it appears that the primitive fibres of the nerves entering into the skin do not lose themselves there *among or in blood-vessels*, as Ehrenberg suspects; nor do they end, as Treviranus thinks, in cutaneous papillæ; nor do they even, as Valentin affirms, and which is really the case in regard to the muscular nerves, go back to their parent stem after having formed terminating loops; *but after they have separated from their parent stem in thicker or thinner bundles, rarely quite single, they form with each other, and with similar bundles of other cutaneous nerves, by alternately uniting and separating, a very close multiplied network; and after that pass immediately into other cutaneous nerves, in order to return with these back to their central organ.*" (p. 47.) See fig. 5.

The following is Burdach's comparison between the mode of distribution and termination of the nerves, in the skin and in the muscles.

1. The cutaneous nerves, immediately on their entrance into the skin, divide into several branches; the muscular nerves, on the contrary, first run a certain distance in the muscles before their division begins.

2. The branches of the trunk of the cutaneous nerve immediately diverge towards different sides, and even their farther subdivisions follow

* Burdach makes use of the words, *branches, twigs,* and *ramuscules,* not in reference to their *comparative thickness,* but only in reference to their relation to the parent stem. The expressions "coming off," "joining," and the like, as applied to branches, twigs, &c., are not to be taken in an absolute sense; for the *branch, twig,* or *ramuscule,* which is described as coming off from a stem, branch, or twig, may in reality as well be said to join it.—Rev.

no definite direction. In the distribution of the muscular nerves, on the contrary, a general direction corresponding to the muscular fibres predominates; although individual branches run across or obliquely over the muscle, in order to spread over the whole breadth of it.

3. The cutaneous nerves, by splitting and ramifying, give off fasciculi which unite with each other and with parts belonging to other cutaneous nerves. These fasciculi form a very multiplied plexus, equally spread over the whole surface of the skin, and representing here and there very regular figures. The muscular nerves form a similar plexus, the so-called *terminating-plexus;* but it is not spread equally over the whole muscle, it is limited to a part of it only: in this place there is also a predominating longitudinal direction, so that it represents only oblique-angled meshes.

4. The individual primitive fibres of the cutaneous nerves, followed through the cutaneous network, pass into another cutaneous nerve, and in this back to its central part; the primitive fibres of the muscular nerves, on the contrary, after emerging from the plexus, return, forming a loop, back to their stem, branch, or even twig.

Valentin has described the loop-like termination of the nerves in the iris and ciliary ligament, and even in parts exclusively sensible; as in the follicle of the teeth, in the skin of the frog, in the interior of the cochlea of birds, in the ampullæ, and in the sack of the vestibule. Breschet has also described the auditory nerve as presenting a loop-like termination; whilst, according to the observations of Wharton Jones, the termination of the auditory nerves resembles more that of the optic.*

A principal result of Valentin's investigations into the mode of termination of the nerves is the doctrine "that the nerves have, properly speaking, no peripheral termination; but that, in their peripheral organs, the centrifugal part passes without any definite change into the centripetal." "This doctrine," says Burdach, "has been proved by Valentin's investigations principally in regard to the motor nerves, and by my own observations in regard to the sentient nerves also of the spinal marrow." He adds, that this principle will probably be found to hold good in regard to all other nerves and in all other peripheral organs; but, as the finest blood-vessels in every organic tissue have a peculiar and characteristic distribution, so surely will the distribution of the nerves within each organ be peculiarly arranged.

The results of Burdach's investigations into the distribution of the nerves in the tongue and mucous membrane of the mouth, (in the frog,) are as follows:

1. The hypo-glossal nerve is distributed to the muscles, and that quite like other muscular nerves, forming plexuses and loops; but differs from them by its ramifications coming off only on one side, and has, with most of the other cerebral nerves, this in common, that the stems of the two sides are not united together by their ramifications.

2. The twigs corresponding to the lingual nerve of the fifth pair belong to the mucous membrane of the mouth and the posterior part of the tongue, and exhibit in these parts an arrangement very similar to that of the cutaneous nerves. They differ from the cutaneous nerves, however,

* Cyclopæd. of Anat. and Phys., vol. ii. pp. 541, 542.

inasmuch as a greater part of their primitive fibres, after a course of a shorter or longer distance from the stem, return to it, (the transition into the stem of the other side Burdach has not yet completely proved); and, moreover, as they never appear to split into single fibres; and, lastly, as they form here and there small knots, which Burdach conjectures to be ganglions.

3. The glosso-pharyngeal nerve of the frog passes through the muscular structure of the tongue, without giving any branches to it, and without forming any plexus. It forms, close at the surface of the tongue and its mucous membrane, a plexus, composed of its finest ramuscules, remarkable for the looseness with which the primitive fibres lie alongside each other. The nerve resolves itself, lastly, into its elementary cylinders which run quite isolated and form terminating loops.

In regard to the functions of the hypo-glossal, lingual, and glosso-pharyngeal nerves, Burdach remarks:

"If we suppose the degree of sensibility of a part to depend in general on the peripheral end of a nerve, it cannot be doubted that a nerve splitting into its finest elementary parts, must manifest greater sensibility than another in which the primitive fibres remain in thicker bundles; so that the nerve, forming an extended network, but one not composed of the finest elements, must be less adapted for special sensation. If we compare, therefore, under this point of view, the very fine ramifications of the optic nerve in the retina, on the one hand, and the so-extended network, but in thicker bundles, of the cutaneous nerves, on the other, it would not be too bold to say that, considering the above-described morphological formation of the glosso-pharyngeal nerve, it is a pure nerve of sense; and that, considering the formation, on the contrary, of the twigs representing in the frog the lingual nerve of the fifth, that it is a nerve of common sensation; and, if we add, moreover, that the hypo-glossal, considering its form, so similar to other muscular nerves, must be a muscular nerve, so would it be proved, by my microscopical investigations of the terminations of the nerves of the tongue, what Panizza has by his experiments physiologically demonstrated." (p. 72.)

We have seen that Remak's observations of the microscopical appearance of the component primitive fibres of the same nerves have led him to a similar conclusion. From Dr. J. Reid's experiments* it would appear that the glosso-pharyngeal is a nerve of common sensation, and certainly not the special nerve of taste.

To Valentin's conclusion, "that the nerves, properly speaking, have no peripheral termination, but that, at their peripheral organ, their centrifugal part passes without any definition into the centripetal," Burdach says he would, in order to make the nature of the termination of the nerves completely evident, add, that "the essential character of all pure nerves of sense consists in this, that they form at their peripheral part a very fine plexus, and resolve themselves into their finest elementary parts; the essential character of nerves of common sensation, moreover, whether they belong to the cerebral or spinal system, depends on this, that they form multiplied, wide-extended plexuses, which consist for the most part of nervous bundles, rarely of single primitive fibres. The essential character of muscular nerves, lastly, is, that within the muscle they form a plexus, partly consisting of thick bundles, and then terminating loops, which very rarely consist of isolated primitive fibres."

That the nerves terminate in the manner above described by Valentin, Emmert, and Burdach is not admitted by all anatomists. Thus,

* Cyclopædia of Anatomy and Physiology, vol. ii. p. 499.)

Treviranus (Heft ii. p. 42,) suspects that, at the terminations of the nerves, the fibres lay aside the sheath in which the cortical fibres are enclosed, to form (as he thinks) a nervous fibre; and that the cortical fibres, thus set free, again separate from each other. This is not the case, he says, with all nerves; but it occurs at some places. These assertions of Treviranus cannot maintain their ground alongside the clear demonstrations of Valentin, Emmert, and Burdach. Müller says, (p. 604,) "it is not very probable that the so-called primitive fibres, which are of considerable size, form the actual termination of the nerves, in parts of which the ultimate elements are much more minute than they. Schwann, indeed, has seen in the mesentery of the frog, issuing from the so-called primitive fibres, numerous finer filaments which here and there presented small knots, from which again several twigs were given off. Further researches on the mode of termination of the nerves in the tail of the larva of the toad have confirmed these observations. The nervous fibrils resulting from the splitting of fibres, of the size of what are ordinarily termed primitive fibres, are excessively minute, and are destitute of the dark tubular sheath which invests the ordinary primitive fibres. The minute knots are almost always present. From the minute fibrils just described, and from the knots on them, still more delicate threads are given off, and terminate by forming a network." On these observations of Schwann, Valentin (*Repertorium*, vol. ii. p. 54,) remarks: "So far as I have been able to observe, these fine fibres have nothing in common with the contents of the nerves, and are merely fibres of cellular tissue, belonging either to the nervous sheaths or accidentally lying beside them. The arched termination of the primitive fibres can be very well observed in the recent conjunctiva of the salamander, in addition to those numerous organs mentioned in my larger work on the nerves."

B. *The Nervous Expansions in which the Optic, Auditory, and Olfactory Nerves terminate.* Ehrenberg first discovered in the retina a tubular structure. According to him, the retina is, in fact, a cerebral substance, formed chiefly by an expansion of the optic nerve covered and penetrated by a close vascular network; in the meshes of which there is found anteriorly a thick layer of free grains, which consist of still smaller granules, and have the greatest resemblance to the globules of the blood.* Ehrenberg describes the substance of the retina as consisting, like the brain, of the so-called jointed or varicose tubular structure in two forms, —viz. 1, of a very fine part, which is, so far as can be made out, *jointed gray substance*; and, 2, of a more distinctly *jointed white substance.* The latter is next the optic nerve itself, of which its fibres are distinctly the continuations. In the retina of the rabbit, the structure consisting of tubes readily assuming the jointed or varicose appearance, is very distinct. The tubules compose, by their aggregation, those two leashes of white streaks seen radiating from the entrance of the optic nerve. The same thing exists, but less distinctly, in the human eye. Ehrenberg mistakes when he says that the tubular structure is in the part which has

* We pass over entirely Ehrenberg's speculations as to the identity between the globules of the blood and those he describes certain parts of the nervous substance to consist of; as also his views regarding the nature of the thymus gland, as being mere conceits.

hitherto been considered the so-called serous layer, or membrane of Jacob. The two parts are quite different: both exist; and each has its own peculiar structure.

In the eyes of some animals, as frogs and fishes, but not in man, Ehrenberg has observed staff-shaped or club-shaped bodies, or papillæ, on the inner surface of the retina; the connexion of which with nerves and vessels, however, remained undetermined. He says, "their connexion with the varicose or jointed tubes of the nerve is not quite clear to me." Ehrenberg says he has convinced himself of the existence of globules in the expansion of the olfactory nerves in the nose, similar to those of the retina. Club-shaped papillæ are found in the olfactory membrane.

Papillæ on the inner surface of the retina are very fully described and well delineated in the work of Treviranus. He considers the tubules as terminating in them. He has observed papillæ in the eyes of all animals. Weber, in a letter to Treviranus, mentions his having seen similar appearances in the human eye twelve hours after death. We have seen the appearance on the inner surface of the retina of the sheep's eye, which Treviranus delineates and describes as papillæ. (See fig. 6.) We have also seen, in the eye of the newt, the structure of the retina exactly as Treviranus delineates it in the frog, in figures 1 and 2 of plate v. Heft iii., and copied in figures 7 and 8 of our plate. Of all animals, he describes the papillæ of the retina of the pike as being the largest. For the purpose of examining the papillæ of the retina, it is necessary to cut off, in a perfectly fresh eye, a small piece of the vitreous humour along with, and still adhering to, the portion of retina to be examined.

The following is a summary of Treviranus's view of the structure of the retina in most mammifera and birds:

"After the optic nerve has penetrated through the sclerotica and choroidea, its cylinders spread themselves out, either singly or in bundles, on the outer surface of the retina in all directions. Each individual cylinder, or each bundle consisting of several cylinders, at a certain part of its course bends in towards the inner surface of the retina. Immediately after this, it goes through openings in a vascular network which springs from the central vein of the optic nerve. Before it arrives at the inner surface of the retina, it penetrates through a second vascular network, formed by the last twigs of the central artery of the optic nerve. After the passage through the latter, it is received by a sheath-like continuation of the vascular layer of the retina, and, covered by this, it terminates behind the vitreous body in the form of a papilla." (Heft ii. p. 52.)

Hence Treviranus explains the greater thickness of the papillæ than of the tubules which terminate in them. He says, (p. 55,) "the cylinders of the auditory nerve and of the olfactory nerve in the mammifera end in papillæ similar to those of the retina, though somewhat more filiform." The papillæ of the auditory nerve Treviranus observed on the spiral lamina of the cochlea, and that most distinctly in young mice. We have seen a similar, but not so defined an appearance of papillæ in the spiral lamina of the sheep's cochlea, as Treviranus delineates on the spiral lamina of the cochlea of a young mouse. (Fig. 4, plate vi. Heft iii., and copied in our fig. 9.) The papillæ of the olfactory nerve Treviranus observed over the spongy bones and partition of the nasal cavities.

In an appendix on the structure of the retina, contained in Heft iii., Treviranus confirms the above, and communicates a great many most interesting particulars regarding the structure of the retina in different animals.

In reference to Treviranus's views, Müller says, "The termination of each separate fibre of the fibrous layer in a staff-like body seems still rather a postulate than an ascertained fact." He adds, "If every nervous extremity corresponded to a fibre of the optic nerve, the thickness of the retina ought to diminish progressively from the point of entrance of the optic nerve to the border of the ciliary ligament, independent of the varying thickness of the coats of the retina." The observations of Gottsche, he says, favour this. The retina of the cuttlefish is a gross illustration of it.

Müller, in his "Jahresbericht," collates an account of the retina of the cuttlefish, by Wharton Jones, published some time ago, with Treviranus's description of the structure of the retina; in such a way that one would suppose he was referring to a description of the retina of the cuttlefish by Treviranus also: but there is none such in the "Beiträge" of the latter, nor do we know if he has anywhere else published on the structure of the retina of the cuttlefish. We quote Wharton Jones's account, as it corresponds in a remarkable manner with the above description by Treviranus of the retina of the mammifera and birds; and is valuable, as the structure in the cuttlefish, though microscopical in some degree, is not so minute and delicate as that in the higher animals. Wharton Jones's account of the retina of the cuttlefish is moreover valuable, inasmuch as it has explained away a great anomaly, which was supposed to exist in the eyes of the cephalopodous mollusca: we mean the supposed existence of a thick layer of pigment on the anterior surface of the retina.

The fibrils from the optic ganglion cover, to a considerable extent, the posterior surface of the eyeball, and each penetrates singly the thin cartilaginous lamina which corresponds to a sclerotica. "The optic fibrils," says Wharton Jones, "having thus entered the eyeball, expand into a layer of a light reddish-brown tinge, which I shall distinguish by the name of the first layer of the retina. What I call the second layer of the retina is the reddish-brown membrane which I have already mentioned is the part usually considered as pigment. It is situated within the first layer; and between the two there intervenes a pretty thick and dark layer of pigment, through apertures in which the nervous substance passes from the first layer of the retina to form the second. Examined with the microscope, the second layer of the retina, which, as I have said, is of a reddish-brown colour, is observed to be composed of short fibres perpendicular to its surfaces. These fibres, towards the inner surface, end in a delicate pulpy nervous substance, also tinged of a reddish-brown colour, particularly on its inner surface, which has a corrugated or papillary appearance."*

The papillæ of the retina of the cuttlefish rise above the thin deposit of pigment which tinges the inner surface; a circumstance which resembles a remarkable peculiarity in regard to the retina of the *Coluber natrix*, described by Treviranus, (Heft iii. pp. 95-96,)—viz. that, on the surface of the retina, turned towards the vitreous humour, there is a

* London and Edinburgh Philosophical Magazine, vol. viii. 1836, p. 2.

black pigment, which "lies between the nervous and vascular layers, admits of being peeled off without injury to the nervous layer, and presents at intervals round transparent places, where the papillæ of the medullary substance, likewise transparent, project."

Volkmann,* Langenbeck,† and Gottsche,‡ have all given descriptions of the retina, agreeing more or less with what has been cited above. It thus appears that the component elements of the retina are tubes or fibres, the continuation of the primitive tubes or fibres of the optic nerve, and globules. The component elements of the nervous expansions in the labyrinth are essentially the same.

We shall now conclude our subject by giving the following account of the intimate structure of the retina in the higher animals, by Valentin, (*Repertorium*, vol. ii. p. 252;) which is the most recent, as it is the most definite and exact, of any that has appeared.

The retina consists, according to Valentin, of three layers, which, proceeding from without inwards, are as follows:

1. The expansion of the primitive fibres.
2. The pavement-like expansion of the globules of the pure overlaying mass, (*Belegungsmasse.*)
3. The layer of proper granules.

The primitive fibres of the optic nerve radiate all over the retina, but with different degrees of distinctness to the naked eye in different animals. The mode of expansion is exactly the same as is the case in regard to the nerves of other parts of the body: that is, the individual nervous stems, or bundles of primitive fibres, do not run simply alongside each other, but interchange their primitive fibres, and thus produce a plexus. The meshes of this plexus have the common character, that they are elongated and pointed at the two ends.

The primitive fibres of the nervous expansion of the retina are remarkable for their thinness. "That they also end in the usual loops is," says Valentin, "considering all their characters, scarcely to be doubted, although their great delicacy renders it impossible to demonstrate this." We mentioned above, in regard to the termination of the auditory nerves, that it resembled more that of the optic nerves than of any other; and we adduced the observations of Wharton Jones in opposition to Valentin's and Breschet's assertions that it has a loop-like termination. We see that Valentin does not venture to assert a loop-like termination of the primitive fibres of the optic nerve in the retina as a thing capable of demonstration: our own observations induce us to consider the primitive fibres of the auditory nerve in the same case.

To proceed with Valentin's description. The middle layer, magnified 240 diameters, and examined with a somewhat shaded light, is seen to consist of whitish, round, granulary globules, arranged by each other in a plane surface. If one of the globules be isolated, and examined under a stronger magnifying power, it is found to consist of an external transparent coat, granulary contents, a transparent vesicular nucleus, and of

* Neue Beiträge zur Physiologie des Gesichtssinns.—Leipzig, 1836.

† De Retina Observationes anatomico-pathologicæ.—Göttingen, 1836.

‡ Ueber die Retina; in Pfaff's Mittheilungen aus dem Gebiete der Medecin.—Altona, 1836. Heft iii. and vi.

a simple kernel enclosed in this. These globules lie under the primitive fibres, and fill up the meshes formed by the latter. (See fig. 11.)

The third layer is the part which has hitherto been always described as the retina: we should mention, it is the layer of papillæ described by Treviranus. It consists of granules, which appear round when slightly magnified, but, under a power of 300 diameters, they appear angular. They are coloured yellow, and contain a denser nucleus-like part in the centre. They lie close together, but are not immediately joined to each other, and are only loosely connected to the middle layer. They form, at the least, looped continuations of the primitive fibres of the optic nerve. (See fig. 10.)

Valentin says, (p. 256,) "Abstracting the peculiar layer of blood-vessels and cellular tissue from the innermost granulary layer of the retina, we have the most complete analogy of conformation with the auditory and olfactory nerves. These also form, by their stems and twigs, plexuses, the meshes of which are of a rhomboidal form, pointed at the two opposite ends, at first more spindle-shaped, and contain between themselves overlaying globules (*Belegungskugel*,) in great numbers. The optic nerve consists of very thin and fine primitive fibres; so also the retina. The retina possesses in the innermost layer a peculiar structure.* (Fig. 10.) This granular layer is wanting at the entrance of the optic nerve, which is a circumstance worthy of notice; for, in consequence of it, this point, being still like pure optic nerve, (only with numerous overlaying globules,) is merely a light-conductor, not an organ impressible by light.

We have thus endeavoured to lay before our readers as clear and complete an exposition as possible of our present knowledge of the intimate structure of the brain and nerves. It will be seen that whatever has any pretensions to exactness has been all accumulated within these five years; and for it we are almost wholly indebted to the labours of German anatomists. Ehrenberg struck out the path, and many have been found to beat it down.

It is commonly considered that the more accurate and complete our knowledge of anatomy is, the more accurate and complete will be our pathology and physiology. This is quite true as a general axiom; but, when we descend into particulars, we find that it does not hold in many instances; for there are parts the anatomy of which we know well, but are still only partially acquainted with their pathology and physiology. Again, there are parts the anatomy of which we know pretty well, and the physiology also pretty well, and yet our knowledge of the latter has been gained in some degree independent of our knowledge of the former. Such is the case with the nervous system. Notwithstanding the great strides recently made in our knowledge of the intimate structure of the brain and nerves, that knowledge has added little to the physiology. The latter, in fact, has been founded on other than mere anatomical data. With the following remarks on this subject by Müller, as being very apposite, we will now conclude; only remarking, that the independency of muscular irritability of nervous influence, which has been repeatedly advocated in this Review, for reasons drawn from the result of experi-

* The papillary structure of Treviranus.

ment, is also favoured, as far as may be, by the mode of termination of the nerves in the muscles.

"That observers," says Müller, "have not inferred too much from the microscopical relations of the nervous fibres, for the purpose of illustrating the physiology of the nervous system, is a circumstance, in my opinion, to be mentioned only with approbation. Our opinion is further, that the results of microscopical observations will have more influence on the future than on the present state of the physiology of the nerves. Microscopical investigation cannot be carried too far in the domain of micrology. . . . This is the point which we keep in view in our own investigations of microscopical objects; and it is with pleasure we remark that most naturalists are of the same mind. The additions to the physiology of the nerves have, for the most part, been made independently of the more recent progress of microscopical research. The only fact by which microscopical anatomy appears to illustrate the physiology of the nerves is that of the isolated course of the primitive fibres in the nervous stems, branches, and plexuses. Even although it should be established as a peculiarity of the nerves of sensation, that the primitive fibres terminate in loops, still this fact is, as we have already mentioned, without any influence on the theory of the phenomena, as the nerves continue to manifest the same phenomena of sensation, even after their ends have been cut away, and consequently the terminating loops removed. Again, suppose every two nervous fibres form a loop with each other at the surface of the brain, still this circumstance, if the continuation of the nervous fibres should really be discussed, is without influence on the explanation of the known phenomena in the muscular nerves: as a cut muscular nerve continues, when irritated, to manifest the same action on the muscle as when the communication with the brain was entire. The physiology of the nerves must, therefore, be developed in an independent manner. Even the isolated course of the nervous fibres might have been, at least, inferred from the phenomena already known to be manifested by the nerves. As to the peculiarities of the different roots of the nerves, we should never have been led to a knowledge of them by microscopical observation. The facts of reflexion, sympathetic sensation, and motion, have been explained by the phenomena themselves, without the necessity of supposing that, in the brain and spinal marrow, there must be a continuation of fibres. The principle for the advancement of the physiology of the nerves thus remains the same,—viz. experiment on the living nerves. And it is only in this way that we shall distinguish the various peculiarities as regards quality in the uniform disposition of the nervous system; particularly as the facts obtained are, in part, more to be depended on than the results of microscopical investigation have hitherto been."—*Müller's "Jahresbericht" for* 1836, p. xxi. *In Archiv. &c. for* 1837. Heft iii.

*References to the Figures.*

Fig. 1. Isolated primitive fibres or tubules from different parts of the brain; exhibiting the various transitions from *a, a,* the almost perfectly cylindrical, to *b, b, b,* the decidedly varicose form. *c, c.* Tubules crooked by the same cause which produces the varicose appearance.

Fig. 2. Primitive fibres of the nerves. At one extremity the nervous medulla is seen oozing out.

Fig. 3. A coarse diagram, illustrating Valentin's account of the structure of ganglions. Deposits of pigment are seen on one side of the ganglionic globules.

Fig. 4. (From Burdach.) This exhibits the fundamental form of the terminating loops of the nerves in the muscles: *a,* a bundle from the terminating plexus; *b, b, b,* fibres which escape the eye without exhibiting terminating loops.

Fig. 5, (also from Burdach,) represents a piece of the skin from the back of a frog, in which four cutaneous nervous stems, A, B, C, and D, spread out in a net-like manner. *a, b, c,* is a dichotomous nervous bundle, which is chosen for the illustration of the different and indeterminable directions in the course of the nervous fibres; *d* and *e,* two bundles intercrossing, and partly exchanging their fibres; *f,* a bundle which rises perpendicularly on another, and gives off its fibres to the latter in divergent directions; *g,* a square formed by two such perpendicularly rising bundles; *h,* an oblique-angled quadrangle; *i, i,* triangles formed by the meeting of a split bundle with another running horizontally, the fibres of the former running on the latter in different directions; *k,* a square formed by the meeting together of two bundles, split at a right angle; *l* and

*m*, rhombus and parallelogram, formed in a similar manner, only the bundles have split at an acute angle; *n* and *o*, a pentagon and a heptagon, produced by the regular meeting together of several bundles, dividing at an obtuse angle; *p*, a dichotomous bundle, with a rounded angle at the place where it divides: at the angle may be seen the transition of the fibres from one branch into the other; *q*, a small stem, formed by the meeting together of several bundles in the middle of the nervous plexus, and soon dividing again into several twigs; *x*, several cutaneous glands; *y*, trunk of a blood-vessel, with deposits of pigment in its walls; *z*, several deposits of pigment found free in the skin.

Fig. 6. (From Treviranus.) The papillæ of the retina of the eye of the sheep, seen from the inner surface, or that turned towards the vitreous body. Magnified 300 times.

Fig. 7. (From Treviranus.) Piece of the retina of a frog, seen from the outer surface. *m, m.* Dark depressed stripe, from which the very broad and rigid cylinders running on this side proceed. Magnified 300 times.

Fig. 8. (From Treviranus ) Papillæ of the retina of the frog, seen from the side turned towards the vitreous body. The dark spots in the centre of these papillæ appear to be the places at which they are connected with the vascular layer covering the inner surface of the retina. The four undermost rows of papillæ are seen sideways. Magnified 300 times.

Fig. 9. (From Treviranus.) Papillæ of the auditory nerve, on a section of the spiral lamina of the cochlea of a young mouse. *a, a.* Osseous part of the lamina, which is quite covered with conical papillæ lying close to each other. *b, b.* Membraneous border of the lamina, on which there are small papillæ of a hemispherical shape, and placed in rows.

Fig. 10. (From Valentin.) Corpuscles of the peculiar granulary layer of the human retina.

Fig. 11. (From Valentin.) The overlaying globules (Belegungskugel) of the human retina.

## Figures and Tables

**Fig. 1. f1:**
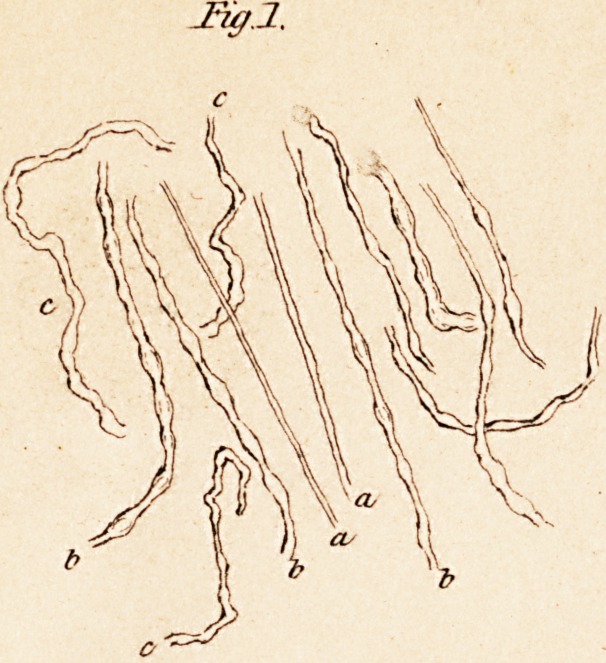


**Fig. 2. f2:**
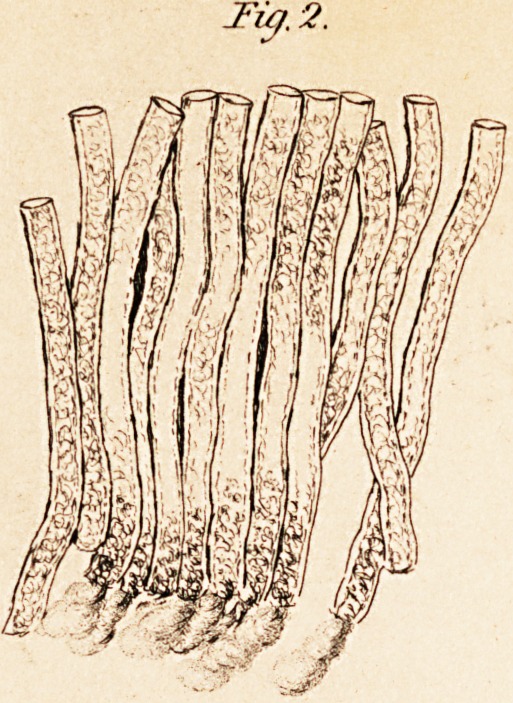


**Fig. 3. f3:**
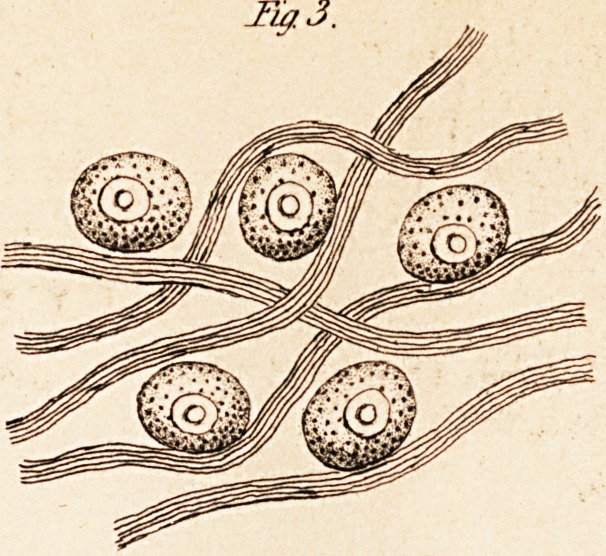


**Fig. 4. f4:**
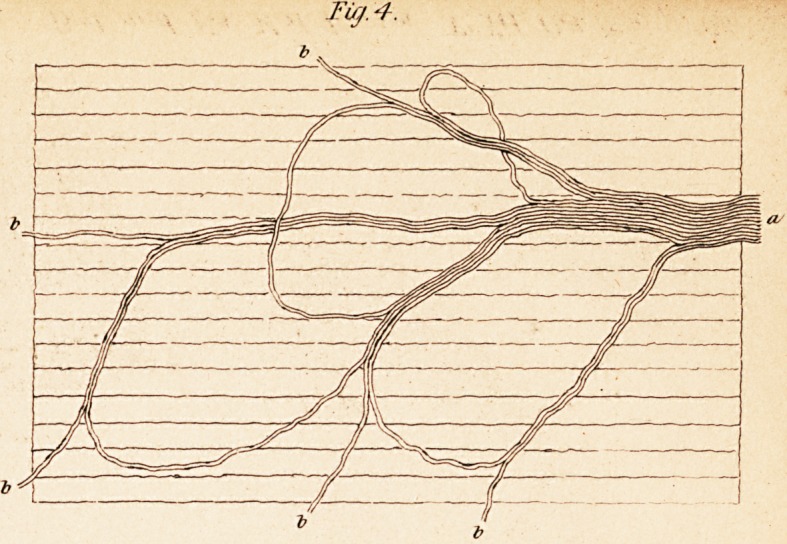


**Fig. 5. f5:**
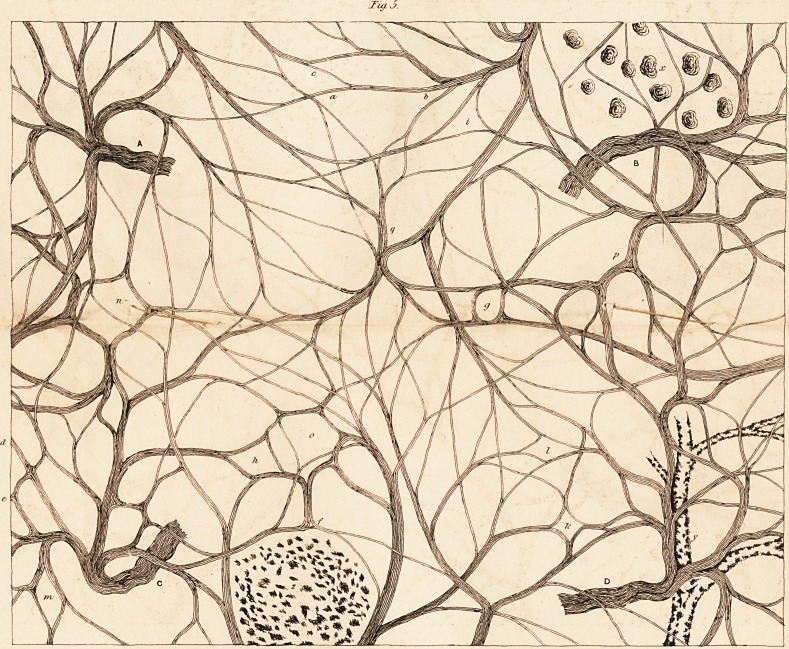


**Fig. 6. f6:**
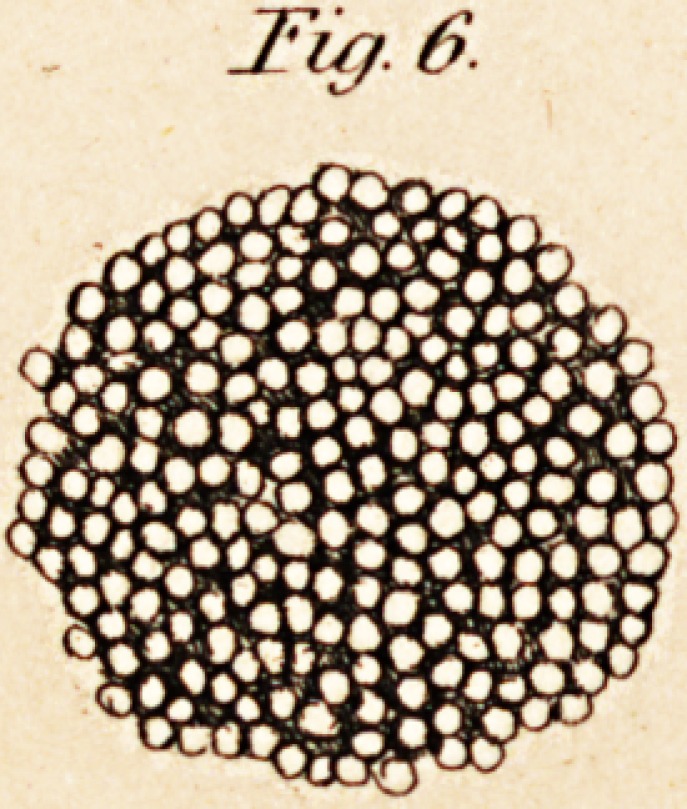


**Fig. 7. f7:**
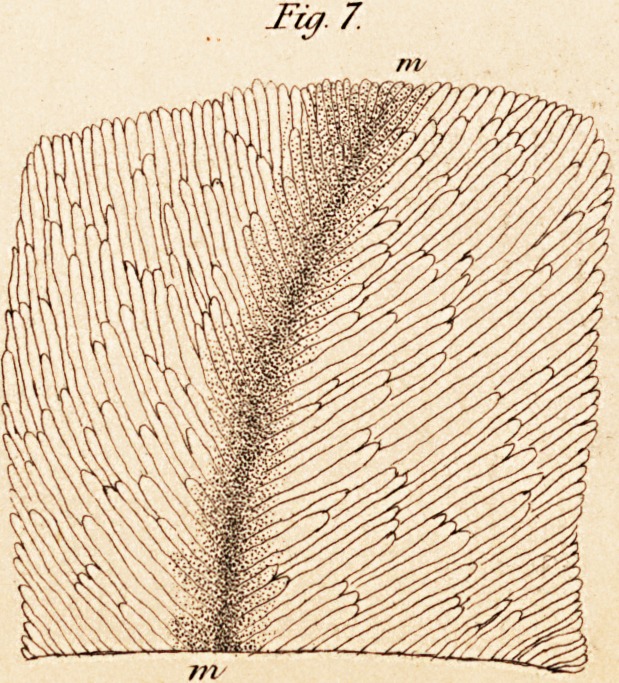


**Fig. 8. f8:**
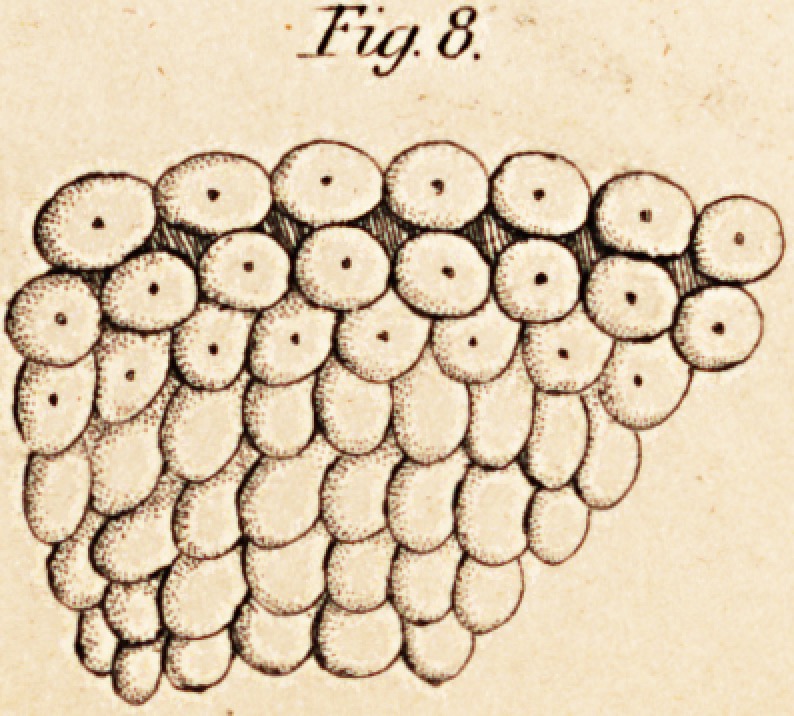


**Fig. 9. f9:**
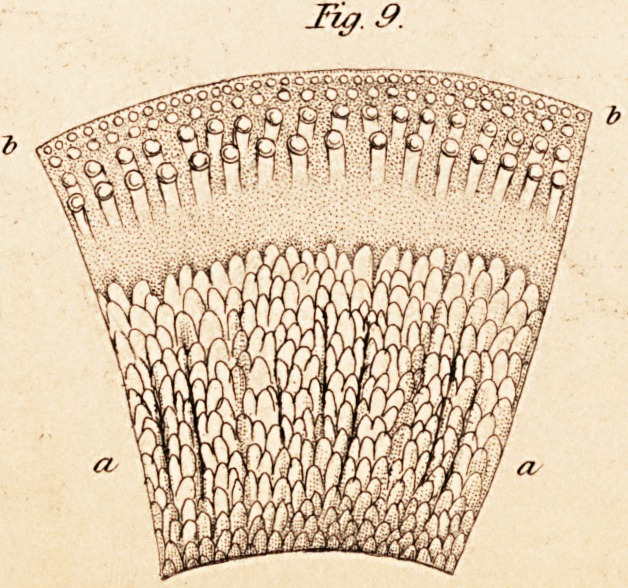


**Fig. 10. f10:**
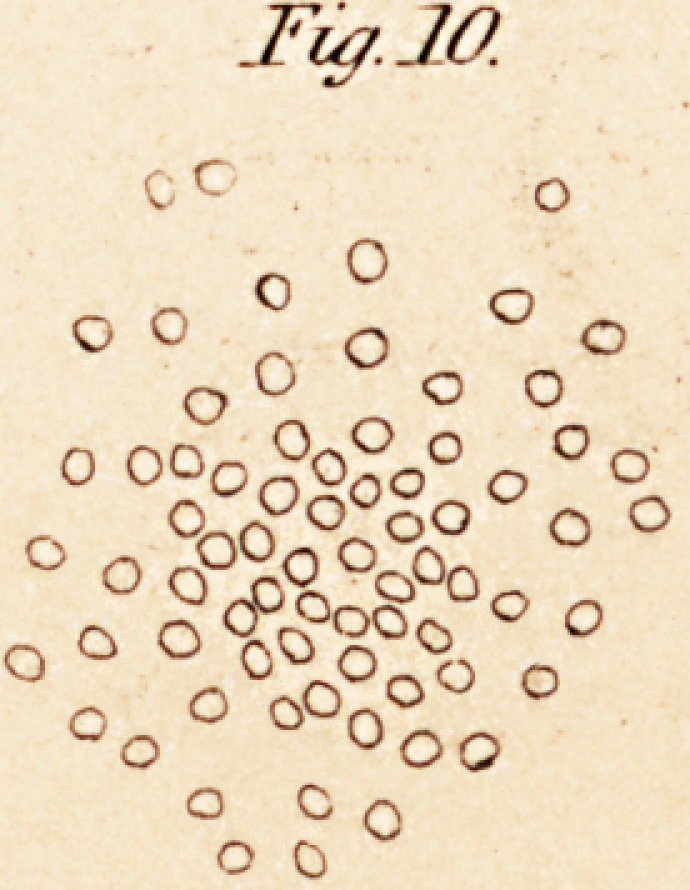


**Fig. 11. f11:**